# Elucidating the cellular determinants of the end‐systolic pressure‐volume relationship of the heart via computational modelling

**DOI:** 10.1113/JP287282

**Published:** 2025-05-11

**Authors:** Francesco Regazzoni, Corrado Poggesi, Cecilia Ferrantini

**Affiliations:** ^1^ MOX, Department of Mathematics Politecnico di Milano Milan Italy; ^2^ Department of Experimental and Clinical Medicine University of Florence Florence Italy

**Keywords:** cardiac physiology, cardiac sarcomere, cross‐bridge kinetics, ejecting contraction, Frank‐Starling law, isovolumetric contraction, length dependence of activation, PV loop

## Abstract

**Abstract:**

The left ventricular end‐systolic pressure‐volume relationship (ESPVr) is a key indicator of cardiac contractility. Despite its established importance, several studies suggested that the mechanical mode of contraction, such as isovolumetric or ejecting contractions, may affect the ESPVr, challenging the traditional notion of a single, consistent relationship. Furthermore, it remains unclear whether the observed effects of ejection on force generation are inherent to the ventricular chamber itself or are a fundamental property of the myocardial tissue, with the underlying mechanisms remaining poorly understood. We investigated these aspects using a multiscale in silico model that allowed us to elucidate the links between subcellular mechanisms and organ‐level function. Simulations of ejecting and isovolumetric beats with different preload and afterload resistance were performed by modulating calcium and cross‐bridge kinetics. The results suggest that the ESPVr is not a fixed curve but depends on the mechanical history of the contraction, with potentially both positive and negative effects of ejection. Cell scale simulations suggest that these phenomena are intrinsic to the myocardial tissue, rather than properties of the ventricular chamber. Our results suggest that the ESPVr results from the balance between positive and negative effects of ejection, related to a memory effect of the increased apparent calcium sensitivity at high sarcomere length, and to the inverse relationship between force and velocity. Numerical simulations allowed us to reconcile conflicting results in the literature and suggest translational implications for clinical conditions such as hypertrophic cardiomyopathy, where altered calcium dynamics and cross‐bridge kinetics may impact the ESPVr.

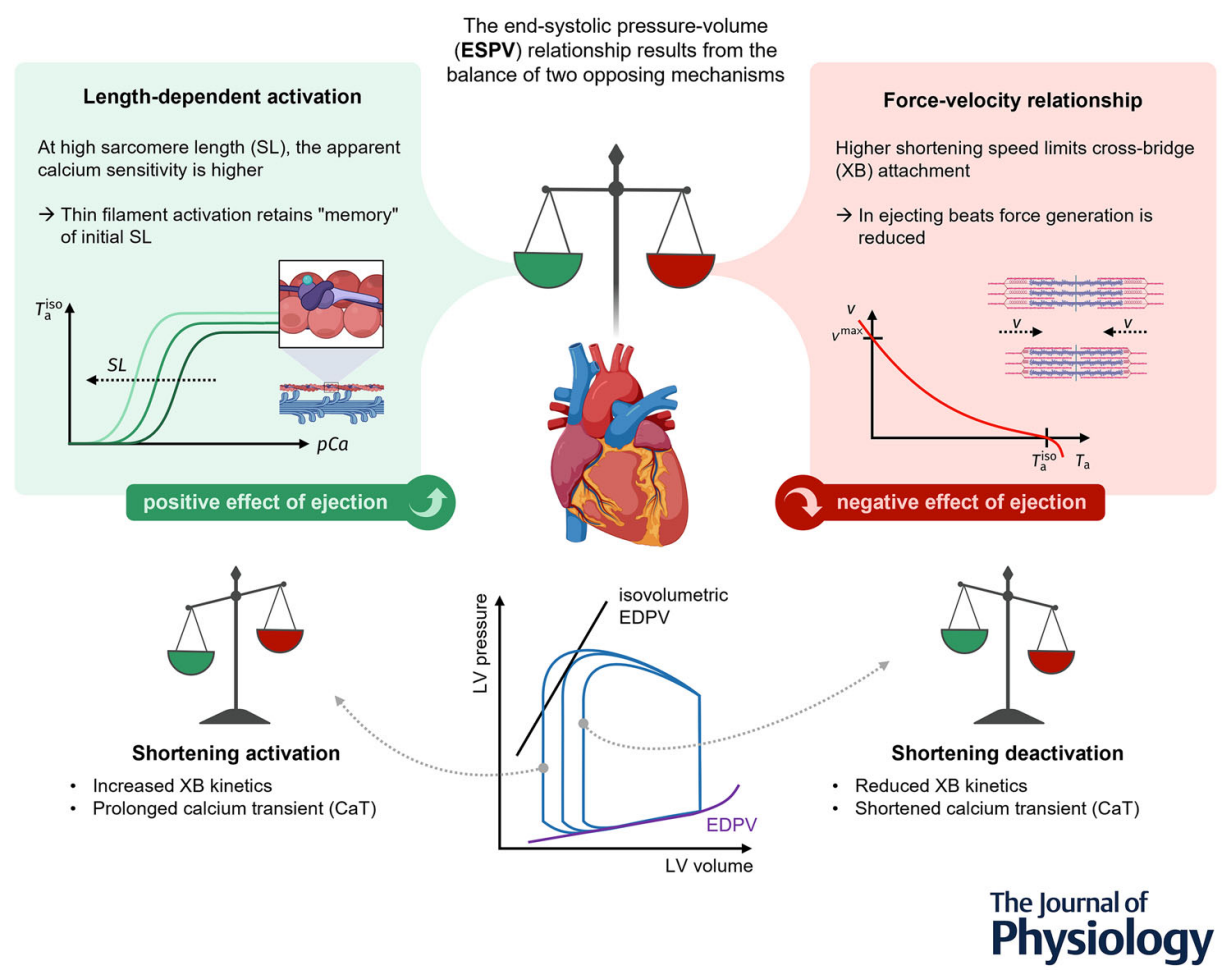

**Key points:**

- The left ventricular end‐systolic pressure‐volume relationship (ESPVr) is a fundamental indicator of cardiac contractility, but the traditional notion of a single, consistent curve across different mechanical modes of contraction (isovolumetric *vs*. ejecting) has been challenged.
- Using multiscale computational simulations, our findings suggest that the ESPVr is not a fixed curve but depends on the mechanical history of the contraction, with both positive and negative inotropic effects during muscle shortening (ejection).
- Our results suggest that these phenomena are intrinsic to myocardial tissue properties, specifically involving calcium kinetics and cross‐bridge cycling, rather than being due to ventricular chamber mechanics.
- Our study reconciles conflicting findings in the literature by providing a mechanistic explanation of how length‐dependent activation and the force‐velocity relationship influence ESPVr.
- This work has potential translational implications for clinical conditions such as hypertrophic cardiomyopathy, where altered calcium dynamics and enhanced cross‐bridge kinetics may significantly affect cardiac contractility and the ESPVr.

## Graphical abstract

**Abstract figure legend** Using a multiscale computational model of left ventricular electromechanics, we investigated how sarcomere dynamics influence the end‐systolic pressure‐volume (ESPV) relationship in ejecting beats compared to isovolumetric beats. Our results indicate that the ejecting ESPV relationship emerges from the interplay of two opposing mechanisms. On one hand, ejecting beats exhibit a memory effect due to the higher thin filament activation in early systole, driven by the increased apparent calcium sensitivity at high sarcomere length (SL), leading to pressure enhancement. On the other hand, fibre shortening limits cross‐bridge (XB) cycling efficiency through to the well‐known inverse force‐velocity relationship, resulting in pressure attenuation. When the first mechanism outweighs the second, ejection has a net activating effect, causing the ejecting ESPV relationship to exceed the isovolumetric one. Conversely, when the second mechanism dominates, shortening has a deactivating effect. In particular the first mechanism is enhanced by a prolonged calcium transient, whereas the second is favoured by reduced XB kinetics.

## Introduction

An essentially complete description of the mechanical dynamics of the heart is given by the changes in pressure and volume of the ventricles during each beat. The end‐systolic pressure‐volume (ESPV) relationship of the left ventricle (LV), obtained by connecting a series of pressure‐volume points at end‐systole in different cycles with different end‐diastolic volumes (EDVs) and different afterload resistances, has been proposed as the best approach to defining cardiac contractility (Suga, 1990).

The first presentation of the ESPV relationships dates back to the end of the 19th century when Otto Frank reported a schematic diagram summarizing his experiments on the frog heart (Frank, 1899). Frank studied different mechanical conditions of contraction (isovolumetric, isotonic with no afterload and afterloaded beats) and adjusted the initial filling volume of the frog's ventricle to different levels. From Frank's schematic diagram (redrawn in Fig. 3 of Sagawa et al., 1990, who published an English translation of Frank's original paper, see also Fig. 1 of Kuhtz‐Buschbeck et al., 2018 for a publicly accessible reproduction) it can be seen that the ESPV relationship of isovolumetric contractions is above that of isotonic contractions (with zero afterload), whereas that of afterloaded contractions is in between. This means that the end‐systolic pressure of the ejecting contractions is lower than that of the isovolumetric contractions initiated at the same end‐systolic volume. Otto Frank thus showed that the relationship between end‐systolic pressure and volume is strongly influenced by the mechanical mode of contraction.

**Figure 1:**
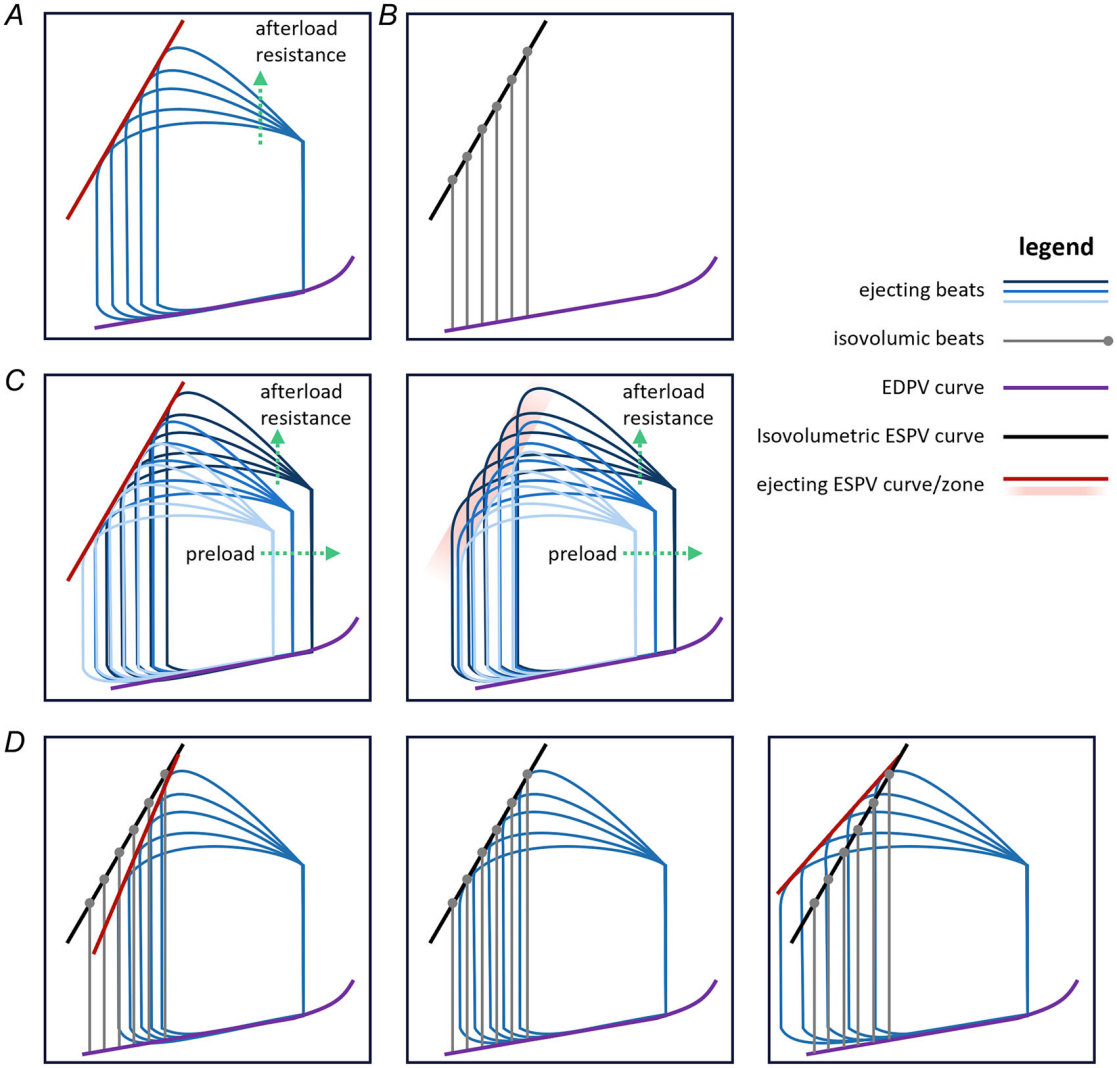
Representation of the end‐systolic pressure‐volume (ESPV) relationship and shortening deactivation/activation *A*, illustration of the ejection ESPV curve obtained for a given end‐diastolic volume (EDV), i.e. the envelope of several PV loops obtained by varying the afterload resistance (we refer here to the resistance that the heart has to overcome during ejection, and not to the pressure that determines the opening of the aortic valve). *B*, illustration of the isovolumetric ESPV curve, obtained by connecting the peaks of the isovolumetric pressure‐volume recordings associated with different volumes. *C*, comparison of two scenarios: whether the ejecting ESPV relationship is represented by a single curve (left) or a zone (right). *D*, comparison of three scenarios: shortening deactivation (left), no shortening effect on the ESPV relationship (centre) and shortening activation (right).

Although Frank's results were later confirmed in various experimental models (for a review see Han et al., 2021), many other studies, including those of Suga and Sagawa in the isolated dog heart (Sagawa et al., 1988; Suga et al., 1973), challenged this view and concluded that the LV ESPV relationship was a single curve relatively insensitive to loading conditions. The slope of this single relationship and its position on the pressure‐volume plane were modified only by changes in cardiac inotropism.

Nevertheless it has long been known that shortening of striated muscle reduces force generation (Edman, 1975; Leach et al., 1980), and, similarly, cardiac ejection has been shown to have a deactivating effect that reduces LV end‐systolic pressure (e.g. Shroff et al., 1985; Westerhof & Elzinga, 1978). These observations and recent studies in isolated cardiac trabeculae that introduced the concept of an end‐systolic zone, rather than a single relationship (Han et al., 2019), are consistent with Frank's original findings and support the idea that the ESPV relationship may depend on the mechanical mode of contraction.

To complicate these conflicting results, previous studies in isolated (e.g. Burkhoff et al., 1993) and *in situ* hearts (e.g. Igarashi et al., 1991) and in isolated cardiac trabeculae (e.g. De Tombe & Little, 1994) have shown that under certain experimental conditions ejection (and shortening) may have the opposite effect to that originally described by Frank. This means that the end‐systolic pressure of afterloaded contractions may exceed that of isovolumetric contractions started at the same end‐systolic volume. Both negative and positive effects of ejection appear to be basic properties of cardiac muscle and not due to properties of the ventricular chamber (De Tombe & Little, 1994).

Despite extensive investigation, it remains debated whether the ESPV relationship is a single curve or a broader region, as does the impact of ejection on contractility.

Given the lack of consensus on the nature of the ESPV relationship, it is useful to establish clear definitions to unambiguously frame the issues under investigation. A commonly used operational definition of ESPV involves constructing the envelope of multiple PV loops, as depicted in red in Fig. 1*A*. Specifically for a given EDV, varying the afterload resistance generates a family of PV loops, all of which are tangent to a single curve. This curve defines the ejecting ESPV relationship associated with that EDV. Conversely, if we consider beats under isovolumetric conditions (i.e. when the aortic valve remains closed), we obtain the isovolumetric ESPV curve by connecting the peak pressure‐volume points recorded at different volumes, as shown in black in Fig. 1*B*.

Now let us return to the ejecting ESPV relationship and imagine repeating this procedure while varying EDV. According to the view that the ESPV relationship is unique (e.g. as proposed by Suga et al., 1973), the PV loops at different EDVs will all be tangent to a single well‐defined curve, as illustrated in Fig. 1*C* (left), which depends solely on cardiac inotropism. An alternative hypothesis, supported by (Han et al., 2019), is that the ESPV relationships at different EDVs define a broader region rather than a single curve – referred to as the ESPV zone, as shown in Fig. 1*C* (right).

A second key question is how the ejecting ESPV relationship (whether a curve or a zone) compares to the isovolumetric ESPV curve. As illustrated in Fig. 1*D*, three possible scenarios can be considered: (1) shortening activation, where ejection enhances contractile force, causing PV loops to exceed the isovolumetric ESPV curve as shown by Igarashi et al. (1991); (2) shortening deactivation, where ejection reduces contractility, causing PV loops to fall below the isovolumetric ESPV curve as originally described by Frank (1899); or (3) no effect of shortening, in which case the ejecting ESPV relationship essentially coincides with the isovolumetric ESPV curve as shown by Suga et al. (1973).

It is important to note that the above two points are independent. The first concerns the fundamental nature of the ejecting ESPV relationship – whether it is a well‐defined curve or a broader region in the PV plane. The second question addresses the physiological effects of ejection on contractility – whether ejection enhances, reduces or has no impact on the ESPV relationship.

The nature of the ESPV relationship and the effects of contraction, as discussed above, have been studied not only at the organ level but also at the tissue level, for example, using isolated trabeculae (Han et al., 2019). In this context the relationship between tension and sarcomere length (SL) is examined instead of the PV relationship. However, quantitatively linking these data to whole‐organ properties is challenging, primarily due to the difficulty of accurately measuring SL *in vivo*. Indeed the working range of sarcomeres *in vivo* is still poorly known. Data obtained in Langendorff‐perfused hearts (Botcherby et al., 2013) may not be able to replicate the *in vivo* conditions, with all the constrains of the organ in its pericardial cavity. Fukuda's lab (Kobirumaki‐Shimozawa et al., 2016) developed a high‐speed (100 frames per second), high‐resolution (20 nm) imaging system to map cardiac sarcomere dynamics in living mice. These authors found broad distributions for both diastolic SL (1.8–2.4) and systolic SL (1.3–2.1 µm), providing valuable insights despite the limitations of extrapolating these findings to human physiology.

Mathematical models were used to investigate the mechanisms underlying the dependence of the cardiac ESPV (or tension‐length) relationship on the mechanical mode of contraction. These models have studied the influence of the sarcomere force‐length dynamics, thin filament inactivation, Ca^2+^‐myofilaments interaction and cross‐bridge (XB) cooperativity on the load dependence of the PV relations. These studies considered either cell scale simulations (e.g. Iribe et al., 2014; Kalda et al., 2015; Landesberg, 1996; Shimizu et al., 2002) or simplified ventricular models, such as the spherical model (e.g. De Tombe & Little, 1994; Pironet et al., 2013). However the mechanisms that can modulate and even reverse the effects of ejection (shortening) on the end‐systolic pressure have not yet been identified.

Disease‐associated variations in XB kinetics and calcium transient (CaT) profile are hallmarks of heart failure with both reduced and preserved ejection fraction (e.g. hypertrophic cardiomyopathy (HCM)). In addition the relative expression of alpha and beta myosin (severely affecting XB kinetics) in human myocardium can vary in life, even in physiological settings. This reinforces the need of a comprehensive mathematical model that includes the cellular and three‐dimensional (3‐D) organ level to predict the effects of these changes in XB cycling and CaT profile.

In this work we used a multiscale computational model of cardiac mechanics to investigate the factors influencing the ESPV relationship and to elucidate the links between subcellular mechanisms and organ‐level function. 3‐D computational models typically neglect the force‐velocity dependence due to numerical instabilities associated with this subcellular mechanism (Augustin et al., 2016; Fritz et al., 2014; Pfaller et al., 2019; Santiago et al., 2018; Strocchi et al., 2020). However using a recently proposed numerical stabilization technique (Regazzoni & Quarteroni, 2021), we were able to highlight the role of the force and velocity in shaping the ESPV relationship. Thus this work demonstrates that a single computational model can predict both positive and negative effects of ejection, depending on the parameters governing the kinetics of subcellular processes. Furthermore we provide a mechanistic explanation for the observed phenomena based on the *in silico* investigation.

## Methods

We considered an *in silico* model of human cardiac electromechanics, based on the iHEART Simulator (Regazzoni et al., 2022), which incorporates a biophysically detailed representation of electrophysiological and mechanical processes, along with their coupling. We considered two simulation settings: cell‐level simulations, which ignore 3‐D effects, and chamber‐level simulations. Our aim was to analyse the impact of shortening on contractility in both settings and to determine to what extent the observed effects are due to intrinsic tissue properties or arise as organ‐level phenomena.

### Microscale electrophysiology model

To model cardiomyocyte electrophysiology, we used the ToR‐ORd model (Tomek et al., 2019), which was developed with the aim of providing a basis for accurate and reliable *in silico* models of cardiac electromechanics. The model has been extensively calibrated and validated using human data (Tomek et al., 2019). In our simulations to avoid artefacts due to the transient associated with the initial condition, we simulated 1000 beats to reach a limit cycle and then used the final state as the initial condition for the simulations. We considered an applied current of 1 ms duration and 53 V/s amplitude as in Tomek et al. (2019). The parameter values reported in the original reference were employed.

### Microscale active force generation model

We considered the RDQ20‐MF model (Regazzoni et al., 2020), which describes the generation and regulation of active force in human cardiomyocytes. The model is based on an explicit description of the major regulatory and contractile proteins. The calcium‐driven regulation of the thin filament takes into account the end‐to‐end interaction of tropomyosin units, thus reproducing the cooperative force‐calcium curves. Although the explicit description of end‐to‐end interactions typically leads to prohibitive computational costs (Rice & de Tombe 2004), the model is formulated as a system of ordinary differential equations by introducing suitable closure relationships. This dramatically reduces the computational cost and allows the explicit representation of cooperative activation in multiscale simulations. The model also accounts for length‐dependent effects on the thin‐filament activation through an effective calcium sensitivity of troponin that increases with the SL based on a prescribed linear relationship, and a maximum tension that depends on the filament overlap. The model has been calibrated using human data, and it is able to reproduce the steady‐state force‐calcium and force‐length relationships, the length‐dependent prolongation of twitches and increase in peak force and the force‐velocity relationship (Regazzoni et al., 2020).

In this work we investigated the effect of the XB kinetics, i.e. the ability of XBs to cycle rapidly, on the ESPV relationship. To do this starting from the baseline parameters of Francesco Regazzoni et al. (2020), we simultaneously rescaled the XB attachment and detachment rates in the RDQ20‐MF model by an appropriate constant, so that the duty ratio, and therefore the maximal force, was unaffected. When the XB rates were rescaled by a constant greater than 1, the kinetics were accelerated, whereas when the rescaling constant was less than 1, the kinetics were slowed. The parameters of the RDQ20‐MF model are presented in Table 1. The value of the XB stiffness parameter $a_{XB}$, which has the effect of rescaling the generated force without affecting the kinetics, was calibrated as described in later sections.

**Table 1:** Parameters of RDQ20‐MF model.

**Parameter**	**Unit**	**Value**		
		**XB kinetics x0.5**	**XB kinetics x1.0**	**XB kinetics x2.0**
μ	‐	10	10	10
γ	‐	12	12	12
Q	‐	2	2	2
$k_{d}$	µM	0.381	0.381	0.381
$\alpha_{k_{d}}$	µM µm^−1^	−0.571	−0.571	−0.571
$k_{\mathrm{off}}$	s^−1^	100	100	100
$k_{\mathrm{basic}}$	s^−1^	13	13	13
$\mu_{f_{P}}^{0}$	s^−1^	12.24	24.49	48.98
$\mu_{f_{P}}^{1}$	s^−1^	0.292	0.584	1.167
$r_{0}$	s^−1^	50.37	100.73	201.47
α	‐	25.184	25.184	25.184
$a_{\mathrm{XB}}$	MPa	64	64	64

List of the active force generation model RDQ20‐MF used in this manuscript for the three scenarios considered: baseline XB kinetics (x0.1), accelerated kinetics (x2.0) and slowed kinetics (x0.5). For the definition of all parameters see Regazzoni et al. (2020).

### Multiscale electromechanical model

For the simulation of the multiscale electromechanics, we relied on the eikonal‐reaction‐mechanics model (Stella et al., 2022). Specifically the propagation of the action potential in the cardiac tissue was modelled by the eikonal‐diffusion model (Colli Franzone et al., 1990; Colli Franzone et al., 2014), suitable for sinus rhythm conditions. The eikonal‐diffusion model incorporates an anisotropic conductivity tensor that reflects the local fibre and sheetlet orientations. This model was coupled with the ToR‐ORd model by applying an electric current, representing the diffusion current to each point of the domain, starting from the activation time predicted by the eikonal‐diffusion model. The calcium concentration obtained from the ToR‐ORd was in turn fed to the RDQ20‐MF model to calculate the active tension generated. The mechanical behaviour of the myocardium was modelled by the elastodynamic equations (Gurtin et al., 2009) within a hyperelastic framework, using the nearly incompressible exponential constitutive law of Usyk et al. (2002). The hyperelastic material law was supplemented with an active stress tensor incorporating the active tension calculated by the RDQ20‐MF model. The local SL and SL shortening/lengthening rates were derived from the tissue deformation and fed back into the force generation model, thus incorporating the effect of tissue strain and strain rate on active force generation.

### Left ventricle simulations

We considered an LV geometry derived from the Zygote 3‐D heart model (Zygote, 2014), representing an average healthy human heart reconstructed from a high‐resolution computer tomography scan, from which we recovered the unstressed reference configuration assuming a pre‐stressing LV pressure of 7.5 mmHg as in Regazzoni et al. (2022). The fibre distribution was generated using the Bayer‐Blake‐Plank‐Trayanova algorithm (Bayer et al., 2012; Piersanti et al., 2021). Electrical activation was induced by localized stimuli at three points of the endocardium, which replaced the electric conduction system. On the epicardial surface we considered spring‐damper boundary conditions to account for the effect of the pericardium and surrounding organs, with the parameters of Regazzoni et al. (2022), whereas at the ventricular base we considered energy‐consistent boundary conditions (Regazzoni et al., 2020). We applied pressure boundary conditions to the endocardium, with the pressure determined in different ways depending on the simulation protocol considered.

We considered two simulation protocols: isovolumetric beats and ejecting beats. In isovolumetric beats to reproduce the experimental condition of clamped valves, we solved the elastodynamic equations under a constant volume constraint. In this case the blood pressure was determined as the Lagrange multiplier that enforces the constraint. Instead during ejecting beats the valves were kept closed until a pressure of 80 mmHg was reached, and then the LV model was coupled with a two‐element Windkessel afterload model, as described in Stella et al. (2022) (see Fig. 2*A*). A second isovolumetric phase was simulated at the end of ejection, i.e. when the volume time derivative reached zero. Finally in the last phase a ramp was applied to return the volume to its end‐diastolic value. We deliberately chose this modelling approach over more complex closed‐loop models (F. Hirschvogel et al., 2017; Regazzoni et al., 2022) to maintain full control over preload and afterload, allowing us to assess their effects directly. In both cases a limit cycle was reached to avoid artefacts arising from initialization. Both protocols were run for different EDVs, different afterload resistances ($R_{ar}$), different XB kinetics and different CaTs. Isovolumetric ESPV curves were obtained by connecting the peak pressure points obtained with isovolumetric beats for different EDVs (see Fig. 2*D*).

**Figure 2:**
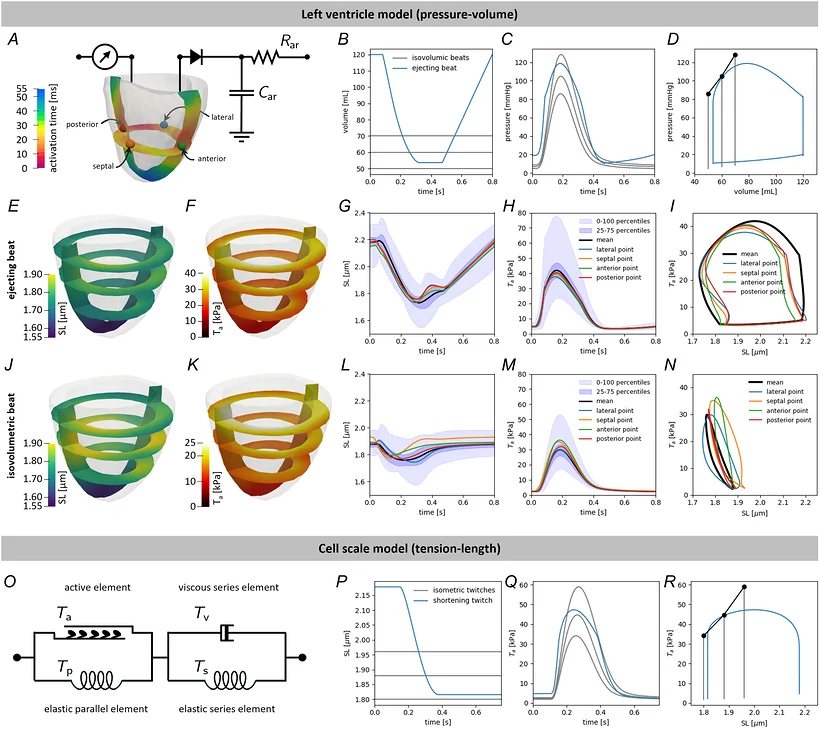
Left ventricle model and cell scale model *A*, illustration of the three‐dimensional (3‐D) left ventricle model, coupled with a Windkessel afterload model, characterized by arterial resistance *R_ar_* and arterial compliance *C_ar_*. The long‐axis and short‐axis views show the activation times obtained under baseline conditions. Four representative points (septal, lateral, anterior, posterior) are shown to serve as reference markers in the upcoming illustrations. *B*–*D*, pressure and volume traces of one ejecting beat (starting with end‐diastolic volume (EDV) = 120 ml) and three isovolumetric beats (with V = 50, 60, 70 ml). In the pressure‐volume plane (*D*) the isovolumetric peaks are connected, showcasing the procedure followed to construct the isovolumetric end‐systolic pressure‐volume relationship (ESPVr). *E*, sarcomere length distribution at time *t* = 0.3 s (corresponding to the end of systole) for the ejecting beat considered in *B–D*. One long‐axis and three short‐axis sections are displayed to highlight the transmural distribution. *F*, active tension distribution at time *t* = 0.3 s for the ejecting beat considered in *B–D*. *G, H*, time traces of sarcomere length and active tension for the ejecting beat considered in *B–D*. The light shaded area represents the range between the minimum and maximum values over space, whereas the dark shaded area shows the range between the first and third quartiles. Black lines represent the space averages, whereas coloured lines correspond to the four representative points displayed in (*A*). Raw data available in the supporting file 3D_model_ejecting_beat_stats.csv. *I*, loops in the tension‐length plane (ejecting beat) associated with average values over space (black line) and with the four representative points displayed in (*A*). Raw data available in the supporting file 3D_model_ejecting_beat_points.csv. *J*–*N*, same as for *E–I*, but this time considering the isovolumetric beat with EDV = 60 ml represented in *B–D*. Note that in *E* and *J* the same colour bar is employed to represent the sarcomere length, in *F* and *K* a different range of active tension is considered due to the highly different tension peaks. Raw data available in the supporting files 3D_model_isovolumic_beat_points.csv and 3D_model_isovolumic_beat_stats.csv. *O*, representation of the single‐cell model, coupled with a parallel elastic element and with a viscoelastic series element. *P*–*R*, tension and length traces of a shortening twitch (starting from SL = 2.18 µm) and three isometric twitches (with SL = 1.80, 1.88, 1.96 µm). In the tension‐length plane (*R‐*), the isometric peaks are connected, showcasing the procedure followed to construct the isometric tension‐length relationship.

The parameters used in this study are presented in Table 2. To select their values we first focused on electrophysiology by setting the conductivity coefficients used in Fedele et al. (2023) and then selecting the parameter c_0_ that guarantees a physiological conduction velocity along the fibre direction (Augustin et al., 2021). Next we manually tuned the passive stiffness of the material, the XB stiffness and the Windkessel model parameters (afterload resistance $R_{ar}$ and compliance $C_{ar}$) to have a PV loop within physiological ranges (Argirò et al., 2022; Bastos et al., 2020; Kawel‐Boehm et al., 2020; Seemann et al., 2019).

**Table 2:** Parameters of the left ventricular (LV) model.

**Parameter**	**Value**	**Unit**	**Description**
**Mechanics**			
ϱ	10^3^	Kg m^−3^	Tissue density
a	0.3256	kPa	Material stiffness
k	18.5	kPa	Bulk modulus
$b_{\mathrm{ff}}$	8	‐	Fibre strain scaling
$b_{\mathrm{ss}}$	6	‐	Radial strain scaling
$b_{\mathrm{nn}}$	3	‐	Cross‐fibre in‐plain strain scaling
$b_{\mathrm{fs}}$	12	‐	Shear strain in fibre‐sheet plane scaling
$b_{\mathrm{fn}}$	3	‐	Shear strain in fibre‐normal plane scaling
$b_{\mathrm{sn}}$	3	‐	Shear strain in sheet‐normal plane scaling
$K_{⊥}^{\mathrm{epi}}$	2 · 10^4^	Pa m^−1^	Normal stiffness at epicardium
$K_{∥}^{\mathrm{epi}}$	2 · 10^5^	Pa m^−1^	Tangential stiffness at epicardium
$C_{⊥}^{\mathrm{epi}}$	2 · 10^4^	Pa s m^−1^	Normal viscosity at epicardium
$C_{∥}^{\mathrm{epi}}$	2 · 10^3^	Pa s m^−1^	Tangential stiffness at epicardium
**Electrophysiology**			
$\sigma_{f}$	2 · 10^−4^	m^2 s−1^	Longitudinal conductivity
$\sigma_{s}$	1.1 · 10^−4^	m^2 s−1^	Transverse conductivity
$\sigma_{n}$	0.55 · 10^−4^	m^2 s−1^	Normal conductivity
$c_{0}$	70	s^−1/2^	Depolarization wave velocity parameter
**Windkessel model**			
$T_{\mathrm{HB}}$	0.8	s	Heart period
$R_{\mathrm{ar}}$	48	MPa s m^−3^	Afterload resistance
$C_{\mathrm{ar}}$	2.5 · 10^−3^	m^3^ MPa^−1^	Afterload compliance

List of the parameters associated with the multiscale three‐dimensional (3‐D) LV model. For their definition see Stella et al. (2022).

### Cell scale simulations

To complement the simulations in the 3‐D LV geometry we also performed simulations at the cell scale. The aim was to loosely mimic the behaviour of individual cells during a heartbeat, but without the 3‐D chamber configuration, to investigate to what extent the results are tissue specific or organ‐level phenomena and to allow a more detailed investigation of the underlying mechanisms. Therefore we considered a cardiomyocyte model described by the ToR‐ORd model coupled with the RDQ20‐MF model, but without spatial detail.

Again two simulation protocols were considered, namely isometric twitches and shortening twitches (corresponding to the two protocols considered for the LV). In isometric twitches the SL was kept at a constant *a priori* value throughout the simulation. Shortening twitches, on the contrary, were simulated with the set‐up shown in Fig. 2*O*: the cardiac tissue was modelled by the parallel between a contractile element and a passive elastic element, connected in series with a viscous‐elastic element to mimic the presence of a load against which the muscle contracts. The length of the contractile element was kept constant until the sum of the active ($T_{a}$) and passive ($T_{p}$) tensions associated with the cardiac cell, denoted by T, reached a prescribed threshold $T_{th}$. From that moment on the cell was free to contract and then relax against the viscous‐elastic series element. As we were not interested in the filling phase in this investigation, we did not include a restretch phase in the simulation. We note that unlike in the LV simulations it is not necessary to run multiple cycles to allow the transient effects of the initial conditions to decay. This is because the simplicity of this model ensures that the initial condition is already in a perfect steady state.

The constitutive behaviour of the parallel passive element was modelled by the law derived from human tissue experiments in Land et al. (2017), i.e. $T_{p}\;\left(\lambda \right)=a\left(e^{b\lambda }-1\right)$, where λ is the tissue strain in the fibre direction. The tension generated by the viscous series component was modelled as $T_{v}=-\sigma \dot{\lambda }$, where σ is the associated viscosity, whereas the elastic series component generated a tension equal to $T_{s}=k_{s}\left(\bar{\lambda }-\lambda \right)$, where $k_{s}$ is the series stiffness, and $\bar{\lambda }$ is the strain corresponding to zero force. In summary the model under consideration gives rise to the following differential equation:

$$\sigma \dot{\lambda }+k_{s}\lambda +T_{p}\left(\lambda \right)+T_{a}=\bar{T}\;,$$

where $\bar{T}$ is set equal to threshold $T_{\mathrm{th}}+k_{s}\lambda_{\mathrm{ED}}$, with $\lambda_{\mathrm{ED}}$ being the end‐diastolic strain at hand. This choice ensures that at the switch from the isometric to the shortening phase the forces are in equilibrium. We calibrated the parameters to obtain tension‐length transients similar to those obtained for single cardiomyocytes in LV simulations under baseline conditions (see Fig. 2), thus obtaining σ=15kPa·s, $k_{s}=25\;\mathrm{kPa}$, $T_{\mathrm{th}}=30\;\mathrm{kPa}$.

Similar to the LV simulations both the isometric and shortening simulation protocols were run for different $\mathrm{end}-\mathrm{diastolic}\;\mathrm{SL}\;(SL_{\mathrm{ED}})$, different viscosities σ, different XB kinetics and different CaTs. Isometric end‐systolic tension‐length curves were obtained by connecting the peak tension points associated with isometric twitches for different $SL_{\mathrm{ED}}$ (see Fig. 2*R*).

### Quantification of shortening activation/deactivation

To quantify the contractile capacity and the effect of shortening on the force generated, we used different metrics for both LV and isolated muscle simulations (see Fig. 3). For LV simulations we define $V_{80}^{\mathrm{ejec}}$ as the LV volume at which the pressure returns to the value of 80 mmHg after exceeding it at the beginning of systole in ejecting beats. Similarly we define $V_{80}^{\mathrm{iso}}$ as the LV volume at which the isovolumetric ESPV curve reaches a pressure of 80 mmHg. Obviously the lower the $V_{80}^{*}$, the more effective the contraction. To quantify the effect of shortening on contractility we define $\Delta V_{80}=V_{80}^{\mathrm{iso}}\;-V_{80}^{\mathrm{ejec}}$: a positive (respectively, negative) $\Delta V_{80}$ denotes the presence of shortening activation (respectively, deactivation).

**Figure 3:**
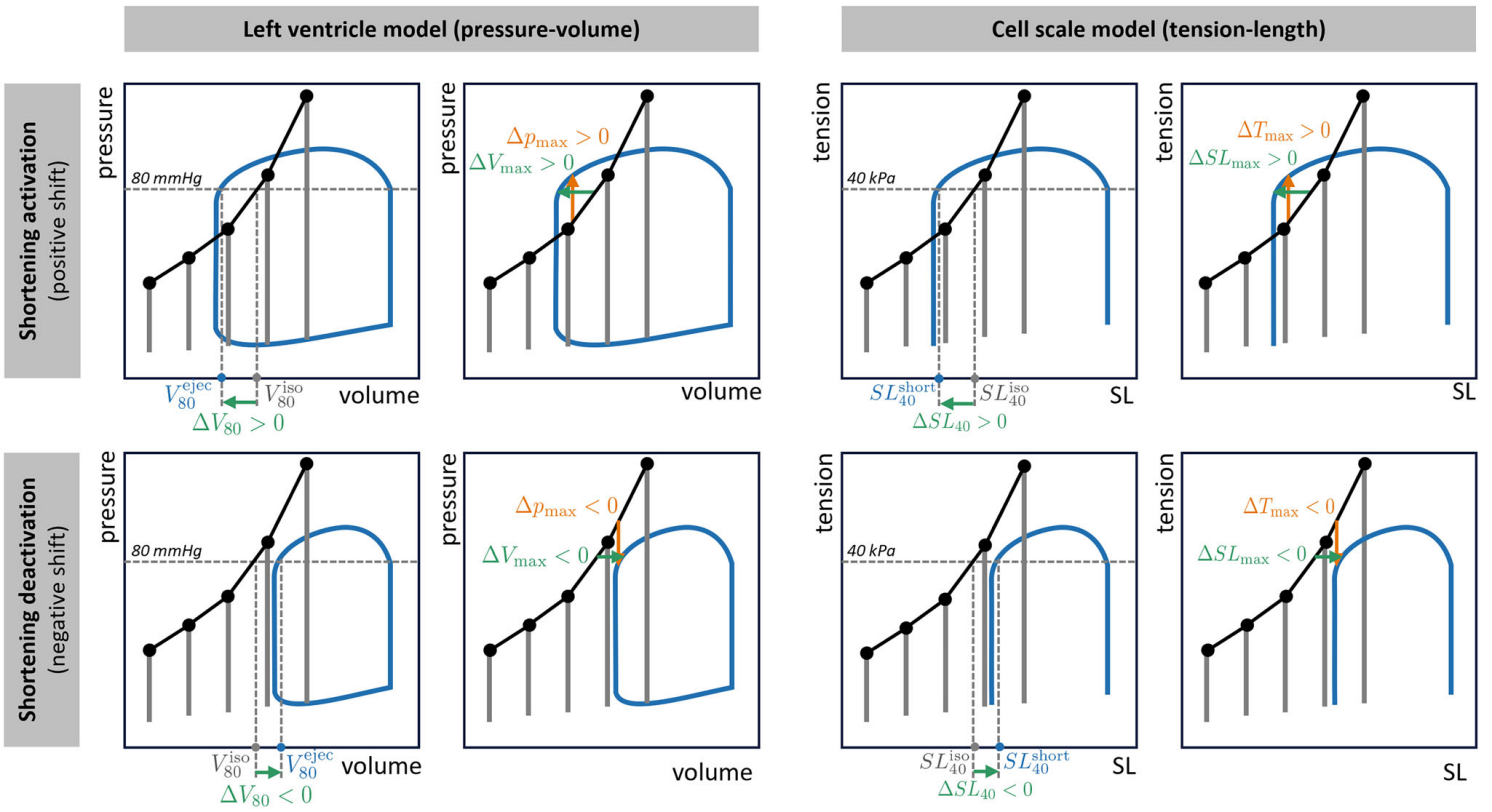
Representation of the metrics used to quantify the impact of shortening upon contractility Left: metrics used in left ventricle (LV) simulations (pressure‐volume plane); right: metrics used in cell scale simulations (tension‐length plane). The first row represents the case of a positive effect of shortening on contractility (shortening activation), whereas the second row corresponds to the case of a negative effect (shortening deactivation). For the LV (or isolated cell) case, consistent with Fig. 2*D* and Fig. 2*R*, the blue lines and labels correspond to ejecting beats (or shortening twitches), the grey lines and labels correspond to isovolumetric beats (or isometric twitches), whereas the black lines represent the end‐systolic pressure‐volume (or tension‐length) relationship. Green labels refer to metrics related to volume (or length), namely $\Delta V_{80}$, $\Delta V_{\max},\Delta SL_{40}$ and $\Delta SL_{\max}$. Orange labels refer to metrics related to pressure (or tension), namely $\Delta p_{\max}$ and $\Delta T_{\max}$.

We note that indices such as $V_{80}^{\mathrm{ejec}}$ are often used in the clinical practice to quantify contractility (Bastos et al., 2020). However from case to case the largest volume shift between the isovolumetric and ejecting case may occur in correspondence of different pressure levels, and not necessarily at $p_{\mathrm{LV}}=$80 mmHg. Based on this observation we define the following two additional metrics:

$$\;\Delta V_{\max}=\max_{t}\left(V^{\mathrm{iso}}\left(p_{\mathrm{LV}}\left(t\right)\right)-V_{\mathrm{LV}}\left(t\right)\right),$$

$$\;\Delta p_{\max}=\max_{t}\left(p_{\mathrm{LV}}\left(t\right)-p^{\mathrm{iso}}\left(V_{\mathrm{LV}}\left(t\right)\right)\right),$$

where $V_{\mathrm{LV}}\left(t\right)$ and $p_{\mathrm{LV}}\left(t\right)$ are the LV volume and pressure transients, respectively, obtained from an ejecting beat. $V^{\mathrm{iso}}\left(p\right)$ denotes the LV volume for which the peak isovolumetric LV pressure is equal to p, whereas $p^{\mathrm{iso}}\left(V\right)$ denotes the peak isovolumetric LV pressure obtained with an LV volume of V. In other words, $\Delta V_{\max}$ and $\Delta p_{\max}$ correspond to the maximum leftward and upward shifts, respectively, in the PV planes between the isovolumetric ESPV curve and the ejecting PV loop (see Fig. 3). Similar to $\Delta V_{80}$ positive values of both $\Delta V_{\max}$ and $\Delta p_{\max}$ indicate an activating effect of shortening, whereas negative values indicate a deactivating effect. The more positive the value, the stronger the activation; the more negative the value, the stronger the deactivation.

For isolated muscle simulations we define analogous metrics by replacing volume with SL and pressure with total tension $T=T_{a}+T_{p}$. Specifically we define $SL_{40}^{\mathrm{short}}$ and $SL_{40}^{\mathrm{iso}}$ as the SL associated with T=40kPa in shortening and isometric twitches, respectively, and we define $\Delta SL_{40}=SL_{40}^{\mathrm{iso}}\;-SL_{40}^{\mathrm{short}}$ as a measure of shortening activation. Then analogous to above, we define

$$\;\Delta SL_{\max}=\max_{t}\left(SL^{\mathrm{iso}}\left(T\left(t\right)\right)-SL\left(t\right)\right),$$

$$\;\Delta T_{\max}=\max_{t}\left(T\left(t\right)-T^{\mathrm{iso}}\left(SL\left(t\right)\right)\right),$$

where $SL^{\mathrm{iso}}\left(T\right)$ and $T^{\mathrm{iso}}\left(SL\right)$ denote the isometric length‐tension and tension‐length curves, respectively.

### Numerical approximation and simulations

To simulate the isolated muscle model we used the staggered time stepping scheme described in Francesco Regazzoni (2020), where the thin‐filament activation variables are updated by an explicit probability‐preserving scheme, whereas the XB variables are updated by an implicit scheme with a coarser time step size. This scheme provides a very good balance between numerical accuracy and computational cost (Regazzoni, 2020). For shortening twitches the parallel‐series elements were coupled with the contractile element through an implicit scheme, relying on the Newton method to solve the associated nonlinear system of equations. Due to the lack of feedback from the contractile model to the ionic model, the solution of the ToR‐ORd model was precomputed, using an adaptive Runge‐Kutta method, and the obtained CaT was provided as input to the RDQ20‐MF model.

For the LV model we performed the spatial discretization using bilinear Finite Elements on hexahedra and relied on the time‐staggered scheme presented in Stella et al. (2022) to couple the submodels. Specifically the solution of the ToR‐ORd model was precomputed as described earlier, and the limit cycle was used in the 3‐D simulation. The microscale activation model and the macroscale mechanics model were coupled using the stabilized scheme recently proposed in Regazzoni and Quarteroni (2021). Remarkably this scheme allowed, for the first time, to remove the numerical oscillations that arise in staggered schemes when the force‐velocity dependence is included in the model, thus allowing the study of the organ‐level effects of this regulatory mechanism, which – as will be shown later – plays a key role in this work. The simulations were performed on the MOX (Dipartimento di Matematica, Politecnico di Milano) cluster computer, using life^x^ (Africa, 2022; lifex, 2025), a high‐performance computing C++ library for finite element simulations of the cardiac function.

## Results

### Baseline conditions

Figure 4 (middle column, labelled ‘XB kinetics x1’) shows the results obtained with the LV model under baseline conditions. The ejecting beats were performed with three different values of EDV (100, 120, 140 ml) and three different values of arterial resistance ($R_{ar}$ = 36, 48, 60 $\mathrm{MPa}\cdot s\;m^{-3}$). As a matter of fact the envelope of all the ejecting PV loops obtained is close to the isovolumetric ESPV curve. However the two curves do not match perfectly: for large EDV, we observe a slight shortening activation effect, as the ejecting PV loops are slightly above the isovolumetric curve; on the contrary for small EDV they fall slightly below the isovolumetric curve, indicating shortening deactivation. Consistently both $\Delta V_{\max}$ and $\Delta p_{\max}$ are slightly positive (respectively, negative) for large (respectively, small) EDV.

**Figure 4:**
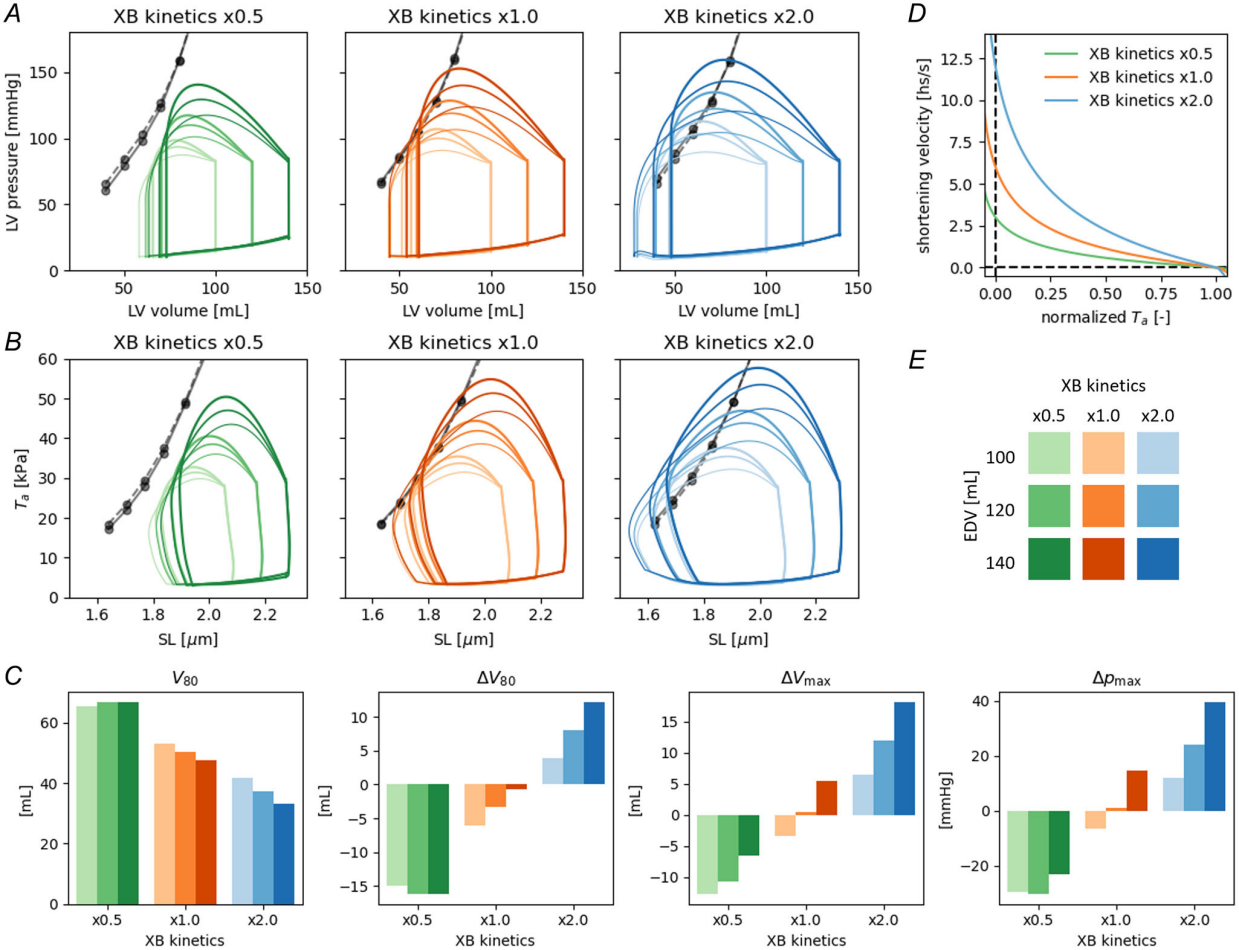
Left ventricle model results for changes in cross‐bridge (XB) kinetics *A*, pressure‐volume loops obtained by varying XB kinetics (corresponding to line colour, see legend in *E*), end‐diastolic volume (EDV) (corresponding to colour brightness, see legend in *E*) and arterial resistance (corresponding to line width, the thinner the line, the lower the resistance – see text for more details). Black solid lines connecting the dots represent the isovolumetric pressure‐volume curve associated with the XB kinetics considered. Black dashed lines instead represent the average pressure‐volume curve among the three XB kinetics considered. *B*, equivalent representation of *A*, showing space‐averaged active tension *versus* sarcomere length instead of pressure and volume. *C*, dependence of $V_{80}$, $\Delta V_{80}$, $\Delta V_{\max}$ and $\Delta p_{\max}$ on XB kinetics and EDV (refer to colour legend in *E*). *D*, force‐velocity relationship obtained from single‐cell simulations for the three levels of XB kinetics considered here (velocity is measured as half sarcomere per second, hs/s). *E*, colour legend. Raw data for this figure available in supporting files 0D_model_isometric_twitch_varying_XBkinetics.csv and 0D_model_shortening_twitch_varying_XBkinetics.csv.

It is noteworthy that the ejecting PV loops are not tangent to a single curve, but rather define an area in the PV plane. This effect is most noticeable for volumes below 70 ml, where multiple curves reach different pressures depending on the preload.

### The impact of XB kinetics

We investigated the impact of modulating the XB kinetics on heart contractility. Figure 4 compares the results obtained under baseline conditions (XB kinetics x1), with the cases where XB rates were accelerated by a factor of 2 (XB kinetics x2) or slowed down by a factor of 2 (XB kinetics x0.5). The results show that an increased (or decreased) XB kinetics has a clear shortening activation (or deactivation) effect. The same conclusion is supported by the trend of $\Delta V_{80}$, $\Delta V_{\max}$ and $\Delta p_{\max}$. In addition in the second row of Fig. 4, we report the space‐averaged active tension and the average SL postprocessed from both ejecting and isovolumetric beat simulations. These results show that the average tension‐length traces closely resemble the corresponding PV traces: the shortening activation/deactivation effect of XB kinetics is already apparent at the cellular level.

To characterize the impact of XB kinetics at the tissue level we present the normalized tension‐length curves obtained on cell scale simulations in Fig. 4*D* when XB rates are rescaled by factors of 0.5, 1 and 2. The figure clearly demonstrates that the faster the XB kinetics, the greater the ability of the tissue to sustain high shortening velocities.

In Fig. 5, we report the results of numerical tests similar to those in Fig. 4 but performed on isolated cells. Specifically we compared isometric end‐systolic tension‐length curves with those obtained from shortening twitches. Shortening twitches were performed by considering three values of $SL_{\mathrm{ED}}$ (2.0779, 2.1783, 2.2765 μM, chosen by taking the mean end‐diastolic SL in the LV tests reported in Fig. 4) and three different viscous damping coefficients, obtained by starting from the baseline value σ=15kPa·s with steps of 3.75kPa·s. The results are in good agreement with those obtained at the chamber level. Specifically an increase in diastolic SL induced shortening activation (slightly more pronounced than in LV tests), and an increase in XB kinetics had a marked shortening activation effect.

**Figure 5:**
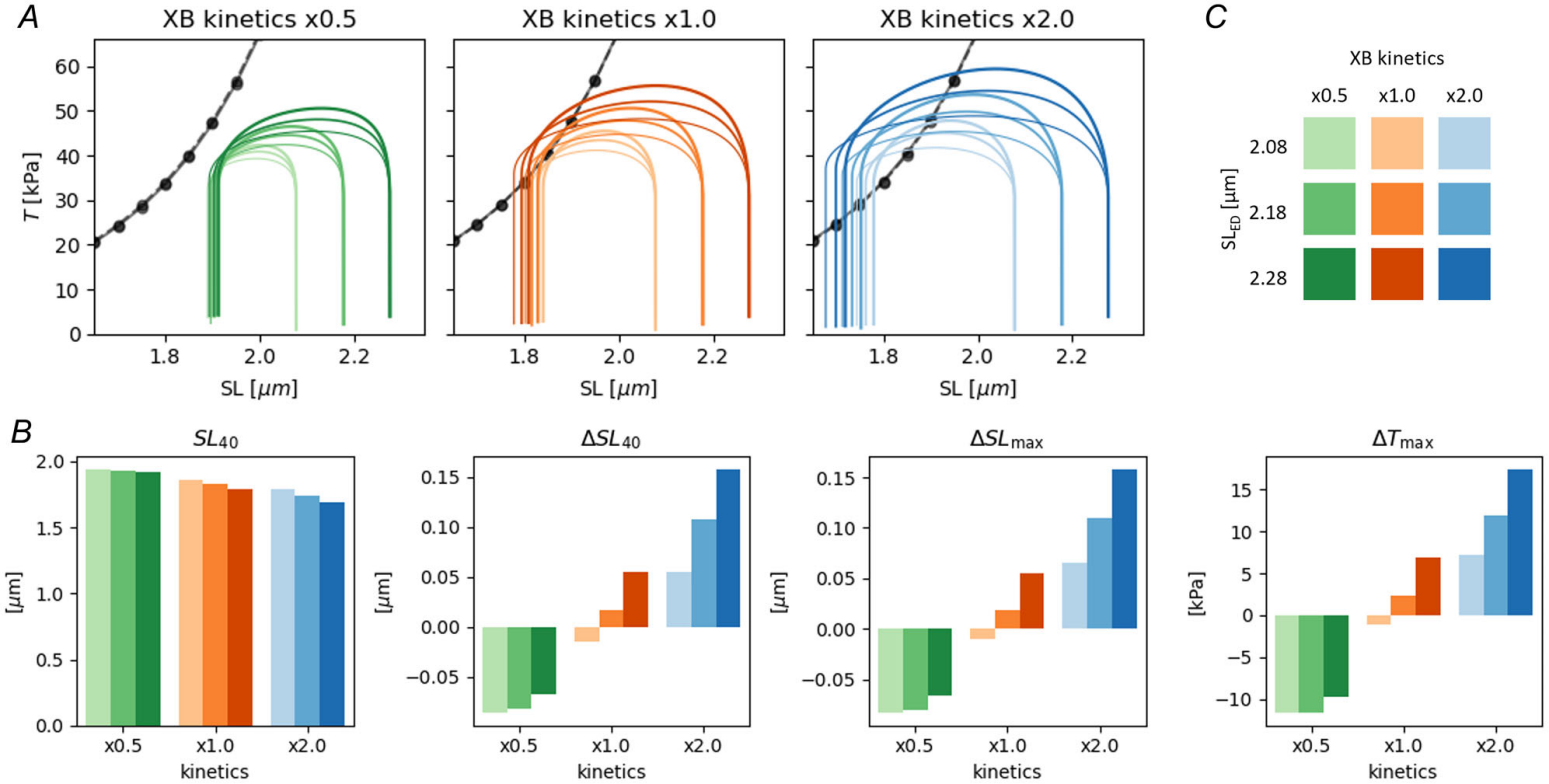
cell scale model results for changes in cross‐bridge (XB) kinetics *A*, tension‐length curves obtained by varying XB kinetics (corresponding to line colour, see legend in *E*), $SL_{\mathrm{ED}}$ (corresponding to colour brightness, see legend in *E*) and viscous coefficients (corresponding to line width, the thinner the line, the lower the viscous damping – see text for more details). Black solid lines connecting the dots represent the isometric tension‐length curve associated with the considered XB kinetics. Black dashed lines instead represent the average tension‐length curve among the three XB kinetics levels considered. *B*, dependence of $SL_{40}$, $\Delta SL_{40},\Delta SL_{\max}$ and $\Delta T_{\max}$ on XB kinetics and $SL_{\mathrm{ED}}$ (see *E* for colour legend). *C*, colour legend. Raw data for this figure available in supporting files 0D_model_isometric_twitch_varying_XBkinetics.csv and 0D_model_shortening_twitch_varying_XBkinetics.csv.

Remarkably in the case of fast XB kinetics it is even more apparent that connecting the end‐systolic points obtained by varying the preload and afterload resistances does not give a unique relationship but rather an area. Similarly in cell scale simulations, the end‐systolic tension‐length relationship defines a region in the tension‐length plane, with a larger area when XBs are accelerated.

### The impact of CaT

To investigate the role of calcium kinetics we accelerated and decelerated the CaT used in baseline conditions by a factor of 20%. Note that the CaTs were adjusted uniformly, so that both the time‐to‐peak and the relaxation time were affected by the same factor. The resulting CaTs are shown in Fig. 6*D*. We repeated the LV simulations, comparing the isovolumetric beats and the ejecting beats obtained with the different CaTs considered (see Fig. 6). First we note that, not surprisingly, the longer the CaT, the higher the LV contractility, as evidenced by the PV loops and the trend of both $V_{80}^{\mathrm{iso}}$ and $V_{80}^{\mathrm{ejec}}$. To quantify the presence of shortening activation/deactivation, we must compare the curves obtained with isovolumetric and ejecting beats for a given CaT. This is done using the indices $\Delta V_{80}$, $\Delta V_{\max}$ and $\Delta p_{\max}$ that consistently indicate that a longer (or shorter) CaT leads to a shortening activation (or deactivation) effect. The same conclusions are drawn from the cell scale simulations (shown in Fig. 7) by looking at $\Delta SL_{40}$, $\Delta SL_{\max}$ and $\Delta T_{\max}$. The impact of CaT duration was smaller when compared to that of XB kinetics reported earlier; however it should be noted that in our investigation we considered milder variations in CaT duration than in XB kinetics, because a too large increase in CaT duration would prevent the LV from fully relaxing, thus rendering the overall simulations uninformative. A more direct comparison of the effects of XB kinetics and CaT duration can be found in the final section of the Results.

**Figure 6:**
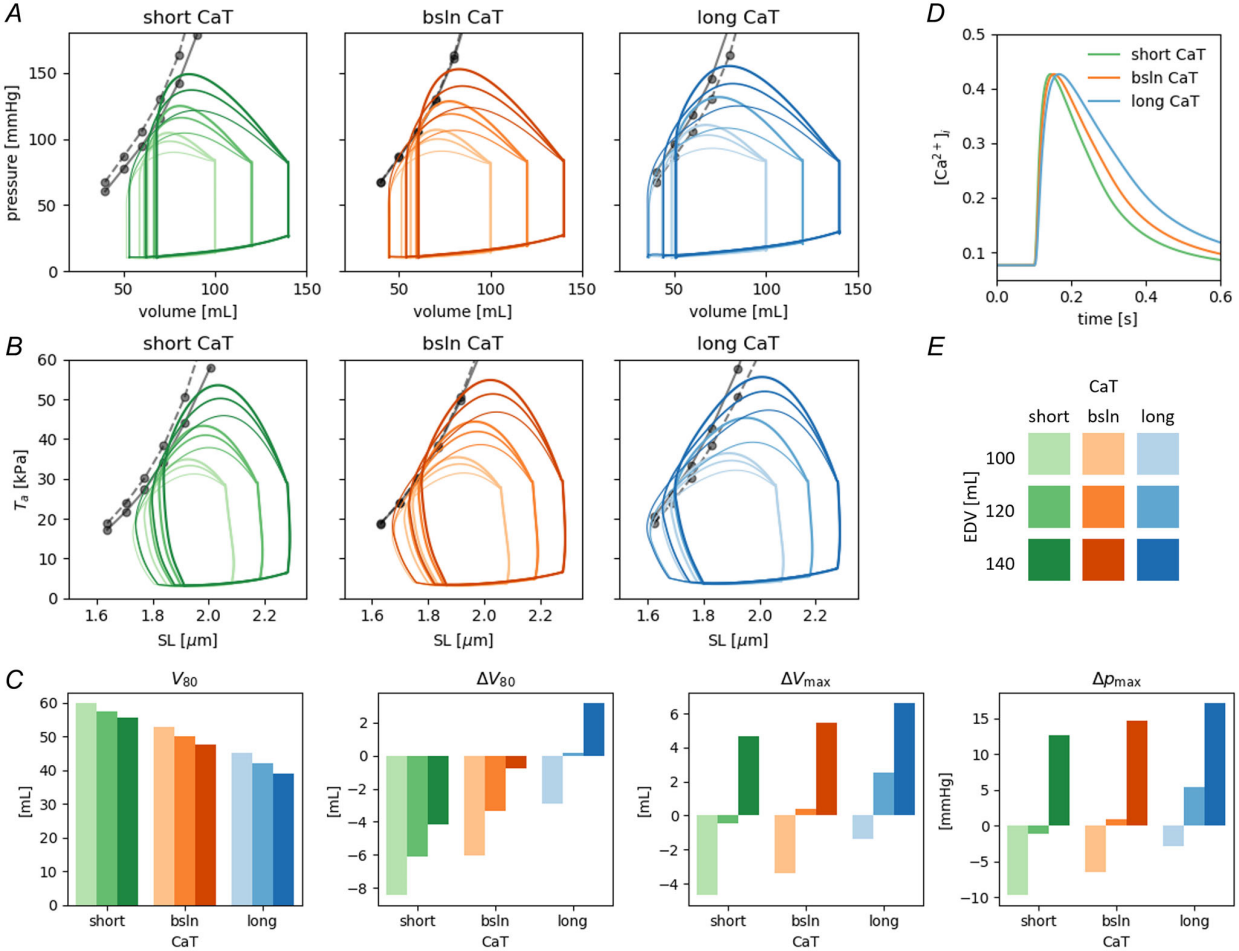
Left ventricle model results for changes in calcium transient (CaT) duration (at constant CaT amplitude) *A*, pressure‐volume loops obtained upon variations in CaT duration with constant CaT amplitude (corresponding to line colour, see legend in *E*), end‐diastolic volume (EDV) (corresponding to colour brightness, see legend in *E*) and arterial resistance (corresponding to line width, the thinner the line, the lower the resistance – see text for more details). Black solid lines connecting the dots represent the isovolumetric pressure‐volume curve associated with the XB kinetics considered. Black dashed lines instead represent the average pressure‐volume curve among the three CaTs considered. *B*, equivalent representation of *A*, showing space‐average active tension *versus* sarcomere length instead of pressure and volume. *C*, dependence of $V_{80}$, $\Delta V_{80}$, $\Delta V_{\max}$ and $\Delta p_{\max}$ on CaT duration and EDV (refer to colour legend in *E*). *D*, CaTs considered in the simulations reported in this figure. *E*, colour legend. Raw data for this figure available in supporting files 3D_model_ejecting_beats_varying_CaT.csv and 3D_model_isovolumic_beats_varying_CaT.csv.

**Figure 7:**
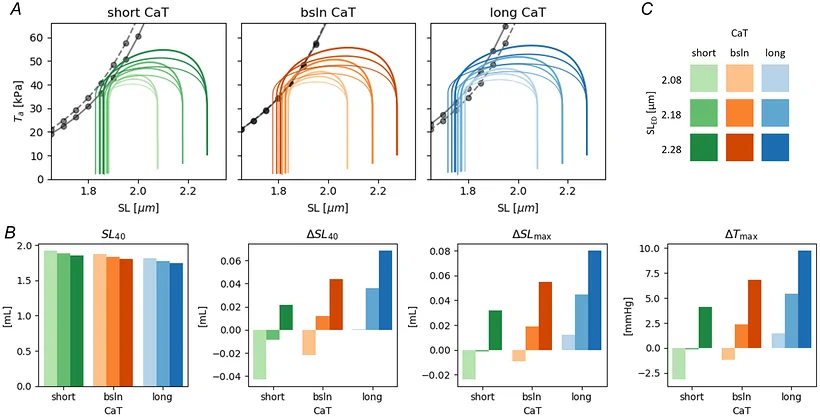
cell scale model results for changes in calcium transient (CaT) duration (at constant CaT amplitude) *A*, tension‐length curves obtained upon variations in CaT duration with constant CaT amplitude (corresponding to line colour, see legend in *C*), $SL_{\mathrm{ED}}$ (corresponding to colour brightness, see legend in *C*) and viscous coefficients (corresponding to line width, the thinner the line, the lower the viscous damping – see text for more details). Black solid lines connecting the dots represent the isometric tension‐length curve associated with the considered cross‐bridge (XB) kinetics. Black dashed lines instead represent the average tension‐length curve among the three CaTs considered. *B*, dependence of $SL_{40}$, $\Delta SL_{40},\Delta SL_{\max}$ and $\Delta T_{\max}$ on CaT duration and $SL_{\mathrm{ED}}$ (see *C* for colour legend). *C*, colour legend. Raw data for this figure available in supporting files 0D_model_isometric_twitch_varying_CaT.csv and 0D_model_shortening_twitch_varying_CaT.csv.

However as noted above, changing the CaT duration not only affects the PV loops of ejecting beats but also shifts the isovolumetric ESPV curve, making it nontrivial to assess the role of CaT duration in determining the shortening effects on contractility. Therefore to decouple the effect of CaT on the isovolumetric contractility from the effect of CaT on shortening activation/deactivation, we performed a second set of numerical tests, in which we corrected the CaT amplitude to compensate for the effects of CaT duration on isovolumetric contractility. Specifically the amplitude of the longer CaT was reduced by 7%, and the amplitude of the shorter CaT was increased by 7% (see Fig. 8*D*). This correction was set to make the isovolumetric ESPV curves almost independent of the CaT considered, allowing a more direct comparison of the three cases in terms of the effect of shortening on contractility. It is noteworthy that the conclusions drawn above still hold even after the amplitude correction: the duration of CaT has a positive impact on contractility during shortening (see Fig. 8).

**Figure 8:**
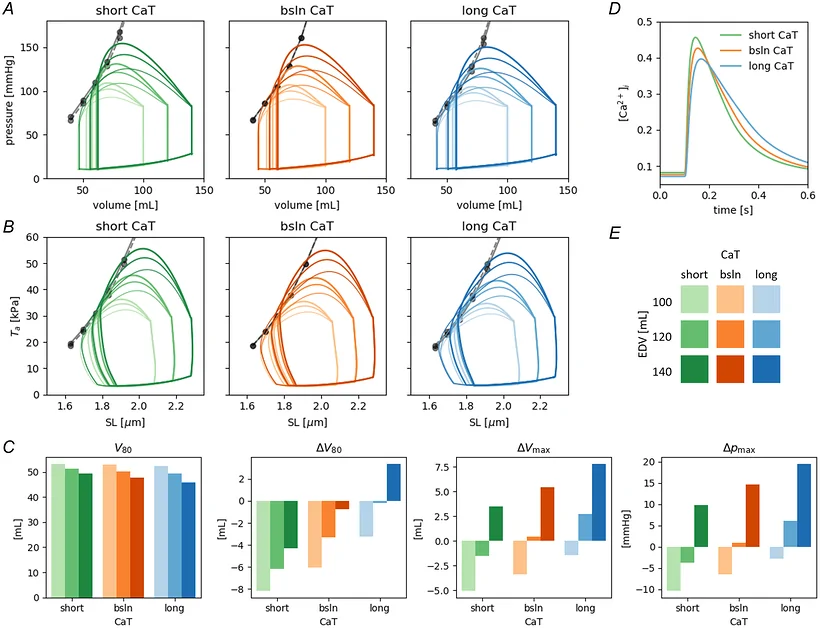
Left ventricle model results for changes in calcium transient (CaT) duration (with CaT amplitude correction) *A*, pressure‐volume loops obtained by varying the CaT duration with amplitude correction as described in the text (corresponding to line colour, see legend in *E*), end‐diastolic volume (EDV) (corresponding to colour brightness, see legend in *E*) and arterial resistance (corresponding to line width, the thinner the line, the lower the resistance – see text for more details). Black solid lines connecting the dots represent the isovolumetric pressure‐volume curve associated with the cross‐bridge (XB) kinetics considered. Black dashed lines instead represent the average pressure‐volume curve among the three CaTs considered. *B*, equivalent representation of *A*, showing space‐average active tension *versus* sarcomere length instead of pressure and volume. *C*, dependence of $V_{80}$, $\Delta V_{80}$, $\Delta V_{\max}$ and $\Delta p_{\max}$ on CaT duration and EDV (refer to colour legend in *E*). *D*, CaTs considered in the simulations reported in this figure. *E*, colour legend.

In Figs 7 and 9 we report the results obtained from cell scale simulations investigating the effects of CaT duration. We considered both the cases of CaT duration modulation with constant amplitude (Fig. 7) and CaT duration modulation with amplitude correction (Fig. 9) by considering the same CaTs used for LV simulations. The results obtained were fully consistent with those of LV simulations: the role of CaT duration on contractility under isometric conditions, as well as the shortening effect on contractility, was the same both at the cell level and at the chamber level.

**Figure 9:**
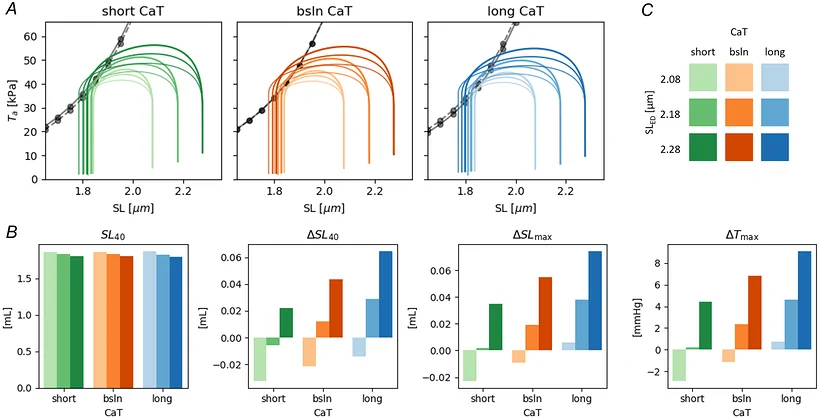
Cell scale model results for changes in calcium transient (CaT) duration (with CaT amplitude correction) *A*, tension‐length curves obtained by varying the CaT duration with amplitude correction as described in the text (corresponding to line colour, see legend in *C*), $SL_{\mathrm{ED}}$ (corresponding to colour brightness, see legend in *C*) and viscous coefficients (corresponding to line width, the thinner the line the lower the viscous damping – see text for more details). Black solid lines connecting the dots represent the isometric tension‐length curve associated with the considered cross‐bridge (XB) kinetics. Black dashed lines instead represent the average tension‐length curve among the three CaTs considered. *B*, dependence of $SL_{40}$, $\Delta SL_{40},\Delta SL_{\max}$ and $\Delta T_{\max}$ on CaT duration and $SL_{\mathrm{ED}}$ (see *C* for colour legend). *C*, colour legend.

### Deeper investigation of microscale variables

Building on the above results suggesting that the shortening effects on contractility are intrinsic to the cardiac tissue, we used the computational model of active force generation to gain mechanistic insight into the underlying cellular basis.

First we considered the cell scale model and performed shortening twitch simulations by varying the XB kinetics. To allow for a finer investigation than that carried out in Figs 3 and 4, we considered milder variations (namely ± 20%). We then performed an isometric simulation by choosing an SL value such that the isometric and shortening tension‐length curves touched at the isometric peak. We then compared the transient of SL, $T_{a}$, and the regulatory units (RU) activation, i.e. the fraction of regulatory units that are in the unblocked state (i.e. they allow XB attachment to the associated actin units). The comparison is shown in Fig. 10*A*. It is noteworthy that RU activation was significantly greater for shortening twitches (with a peak twice as large on average) than for isometric twitches. Increased XB kinetics had a (small) negative effect on the RU activation peak, and – in agreement with the above results – a more pronounced positive impact on the tension peak and on maximum shortening. In addition increasing the XB kinetics slightly decreased the duration of both the RU activation and $T_{a}$ transients.

**Figure 10:**
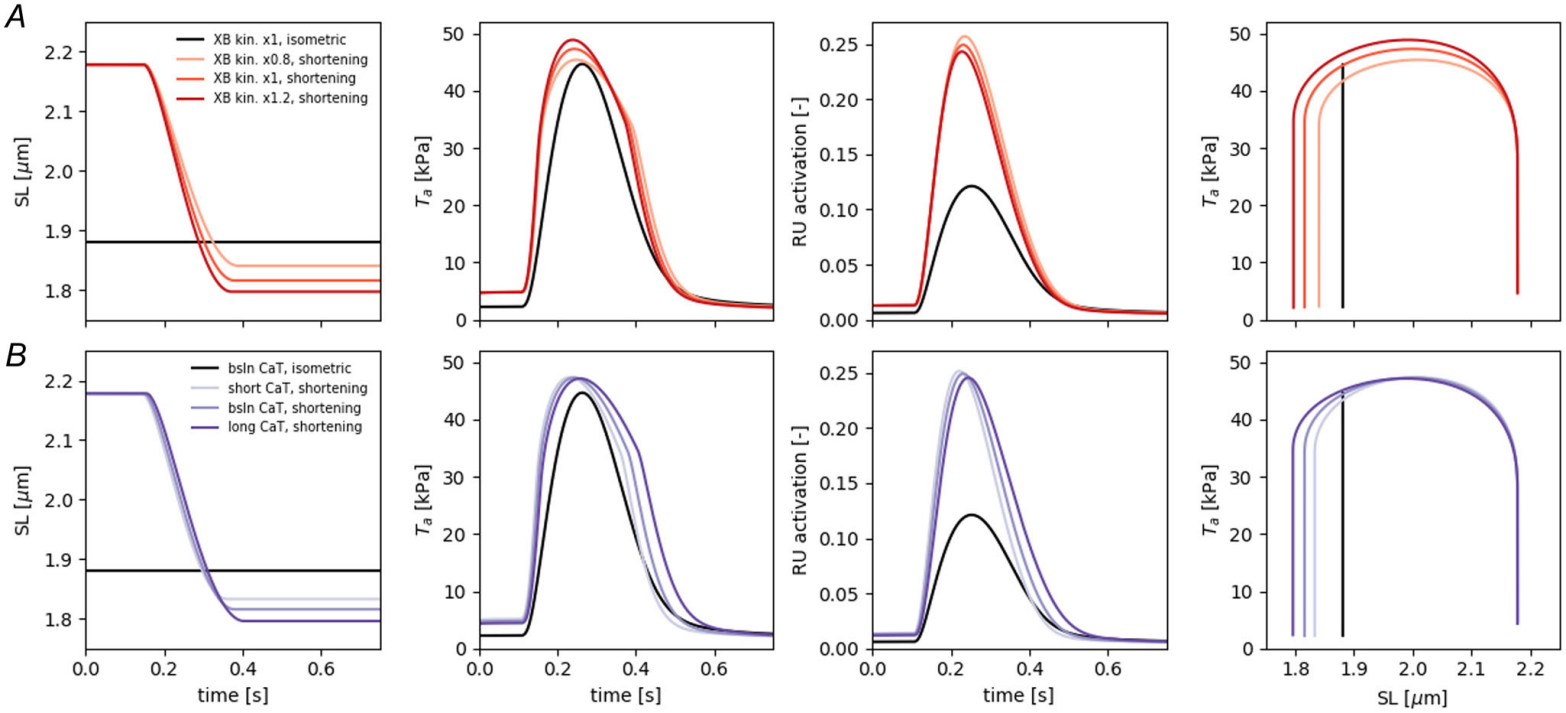
Deeper investigation of microscale variables *A*, traces associated with an isometric twitch and three shortening twitches with ±20% variation in cross‐bridge (XB) kinetics from baseline. From left to right: sarcomere length, active tension, regulatory unit (RU) activation (i.e. the fraction of regulatory units that are in the unblocked state), tension‐length curves. *B*, traces associated with one isometric twitch and three shortening twitches with 20% accelerated/decelerated calcium transients (CaTs) with amplitude correction, as described in the text.

We performed a similar analysis to gain mechanistic insight into the impact of CaT duration. We performed three shortening twitch simulations, with baseline CaT and with 20% accelerated/decelerated CaTs, where we corrected the amplitude to achieve a constant tension peak. We compared the results with an isometric twitch, where SL was chosen so that the tension peak intersected the baseline tension‐length curve (see Fig. 10*B*). Again the shortening twitches achieved a significantly larger RU activation peak than the isometric twitch. Increasing the CaT duration resulted in increased shortening, increased duration of both RU activation and $T_{a}$ transients and a slight reduction in the peak of RU activation.

## Discussion

The computational results suggest that treating the ESPV relationship as a fixed curve is an oversimplification. Instead this study suggests that by connecting the end‐systolic points obtained by varying the preload and afterload resistances, we do not obtain a single curve, but rather a region within the PV plane. This is in line with force‐length recordings on intact trabeculae by De Tombe and Little (1994) and Han et al. (2019), as well as previous numerical studies of both single cells (Iribe et al., 2014; Kalda et al., 2015; Landesberg, 1996; Shimizu et al., 2002) and simplified 3‐D models (Pironet et al., 2013). Furthermore the amplitude of this region may depend on factors such as XB kinetics and CaT. Under baseline conditions the spread of the curves is limited, so that the ESPV region effectively resembles a single curve if the resolution is insufficient. This may explain the traditional view of the ESPV relationship as a fixed curve. However when XB kinetics are accelerated, the model predicts a significantly wider ESPV region, where a single curve approximation would indeed be an oversimplification.

Furthermore this computational study shows that the mechanical mode of contraction (i.e. ejection *vs*. isovolumetric conditions) plays a role in shaping the ESPV relationship. Under baseline conditions the impact was negligible and dependent on the preload, with a small positive effect of ejection for large EDV and a small negative effect for small EDV. However when variations from the baseline conditions were introduced, significant variations in the ESPV relationship of the ejecting beats were observed compared to the isovolumetric curve. This explains the observed variability of the PV loops, which can either exceed or remain below the isovolumetric curve, depending on the specific mechanical conditions and contraction modes, thus reconciling the apparently contradictory findings in the literature, such as those of Frank (1899) and De Tombe and Little (1994). Specifically either an acceleration of the XB kinetics or a deceleration of the CaT leads to an enhancement of generated force during shortening, causing the ejecting ESPV curves to exceed the isovolumetric curve. Conversely either a deceleration of XB kinetics or an acceleration of CaT resulted in ESPV curves below the isovolumetric curve. These findings highlight the complexity of cardiac muscle behaviour and show that contractility is not only defined by intrinsic properties but also by the temporal sequence of activation.

Remarkably despite their simplicity the cell scale simulations showed the same trends as the LV simulations, supporting the hypothesis that the impact of shortening on activation is due to mechanisms that are intrinsic to the tissue, rather than a property of the ventricular chamber. Furthermore both shortening activation and deactivation were obtained as emergent phenomena of the model, as we did not introduce any artificial mechanism to modulate inotropy following the contraction mode. As discussed below, the two ingredients that were crucial to reproduce both phenomena were just the length‐dependent apparent calcium sensitivity of troponin and the force‐velocity relationship.

Motivated by this view, we used cell scale simulations to elucidate the mechanisms governing the effect of shortening on contractility. In fact by simplifying the set‐up to its essentials, the comparisons shown in Fig. 10 allow us to gain insight into the origin of the phenomena observed above.

Let us first compare isometric and shortening twitches under baseline conditions (namely the black and coloured lines in Fig. 10*A*). In the shortening case activation starts at a higher SL than in the isometric case, and, due to the higher calcium sensitivity associated with higher SL, the RU activation increases more steeply. The sarcomere then shortens to, and even falls below, the length assumed in the isometric case. This results into a lower calcium sensitivity than in the isometric case, but the peak RU activation is still higher, as most of the thin‐filament activation process occurred at higher SLs, and thus with a troponin that was more able to bind calcium. This explains the higher RU activation obtained in the shortening case (coloured lines). However looking at the plot of $T_{a}$, we can see that doubling the RU activation does not result in a similar increase in the force generated. The reason for this is that due to the force‐velocity relationship, the force is greatly reduced by shortening. This partially compensates for the increased activation of the thin filament. The conclusion is that the peak force obtained during shortening twitches is the net result of two opposing mechanisms that balance each other: on the one hand, an increased activation of the thin filament, which maintains the ‘memory’ of the higher SL values present during the activation phase; on the other hand, the depressing effect on force due to the speed at which the sarcomere shortens. When the scales of this balance tip to one side or the other, we observe the two phenomena of shortening activation and deactivation described in the literature and also observed in the simulations reported in this paper.

With this in mind let us now consider the effect of changes in the kinetics of XBs. It is well known that the higher the kinetic rates of XBs, the greater their ability to cycle and sustain high shortening velocities without excessive force reduction. Therefore, an increase (or decrease) in XB kinetics reduces (or accentuates) the effect of shortening velocity on the force generated (as also shown in Fig. 4*D*). As a result the second of the two compensatory mechanisms is weakened (or enhanced), leading to an increase (or decrease) in contractility. This is confirmed by the plots of $T_{a}$ in Fig. 10*A*. Consequently increased force generation leads to increased shortening, which, in turn – due to the impact of SL on calcium sensitivity – leads to an, albeit slightly, reduced RU activation, as shown in Fig. 10*A*. However this negative feedback loop does not cancel out the positive effect on $T_{a}$ as it is a secondary phenomenon.

Finally in Fig. 10*B* we examined the effect of changes in CaT duration. In this case, as shown in the figure, the two compensatory mechanisms are not significantly affected. However because CaT is longer, for the same peak force (due to CaT amplitude modulation), a longer $T_{a}$ duration results in more tissue shortening. From another perspective slowing down the kinetics of CaT has the effect of increasing the relative kinetics of XBs compared to CaT, leading to a shortening activation effect due to the mechanism described above.

A limitation of this study is that we have neglected the mechano‐calcium feedback (Levrero‐Florencio et al., 2020; Mazhar et al., 2023), by which the free cytosolic calcium concentration is affected by the calcium‐troponin binding process, potentially leading to differences in the CaT of isometric *vs*. shortening twitches. Although this may alter the results on a quantitative level, we do not expect any significant qualitative changes, and the mechanisms elucidated in this study would remain essentially unchanged. In addition to limit the computational cost, we used the eikonal equation to model action potential propagation, neglecting mechano‐electrical feedback and heterogeneities in action potential duration. However previous studies have shown that as long as the overall electrophysiological activity remains within physiological ranges, these modelling simplifications have a negligible impact on the PV loop (Salvador et al., 2022; Zappon et al., 2024). Another limitation is that we have ignored residual stress and assumed the existence of a reference unstressed configuration, as is common in cardiac computational models (Gerach et al., 2021; Marx et al., 2022; Peirlinck et al., 2018). Finally both isovolumetric and ejecting beats were simulated at steady state without considering cell mechanisms that may be involved in the slow force response to changes in initial cardiac muscle length (Dowrick et al., 2019; Parmley & Chuck, 1973) or in the von Anrep effect (Anrep, 1912; Sequeira et al., 2024).

We now address the potential translational significance of this work, although these aspects were not specifically investigated in this study. It is noteworthy that both an acceleration of XB kinetics and a prolongation of CaT occur in HCM, especially if associated with mutations in myosin binding protein C (Pioner et al., 2023) or beta myosin (Belus et al., 2008; Poggesi & Ho, 2014). The model simulations presented in this paper indicate that this would result in an enhancement of the mechanical performance of ejecting beats, leading to a situation where the ESPV relationship of ejecting beats exceeds the isovolumetric curve. This may contribute to justify the description of HCM as a ‘hypercontractile’ condition. The mutation‐driven increase in XB kinetics is a hallmark of HCM and has never been reported in secondary hypertrophy or in heart failure (HF). Conversely a slowing of CaT decay and, more generally, CaT prolongation is a very common feature of HF across various aetiologies (e.g. ischaemic, hypertensive and valvular defects). Based on our simulations, we can speculate that in HF a prolonged CaT may be an adaptive mechanism to maintain the stroke volume at any given afterload level, despite the reduced force‐generating capacity of the myocardium (e.g. reduced contractility, reduced myofilament density, myocardial disarray).

Another point to consider is the relative expression of alpha and beta myosin isoforms in human myocardium. The rate of XB cycling is 2.5–3 times higher with alpha myosin than with beta myosin (for review see Walklate et al. (2021)). The relative expression of alpha‐ and beta‐myosin heavy chains (encoded by the MYH6 and MYH7 genes, respectively) has been shown to be controlled by thyroid hormones (specifically 3,5,3'‐triiodo‐l‐thyronine, T3) in rodents (Gustafson et al., 1986). Although little is known about the regulation of the expression of the alpha‐MHC gene in the human ventricles, the similarities observed in the 5' flanking region between the rat and human alpha‐MHC genes suggest that the human gene may be regulated in a similar way (Tsika et al., 1990). The acceleration of XB kinetics resulting from a switch from beta‐ to alpha‐myosin isoforms may enhance force generation during shortening, thereby contributing to the hyperdynamic conditions associated with hyperthyroidism.

## Additional information

## Competing interests

None of the authors has any conflicts of interests.

## Author contributions

F.R.: conceptualization, formal analysis, funding acquisition, investigation, methodology, software, visualization, writing – original draft, writing – review and editing. C.P.: conceptualization, funding acquisition, methodology, writing – original draft, writing – review and editing. C.F.: conceptualization, funding acquisition, methodology, writing – original draft, Writing – review and editing.

## Funding

F.R. has received funding from the project PRIN2022, MUR, Italy, 2023–2025, P2022N5ZNP ‘SIDDMs: shape‐informed data‐driven models for parametrized PDEs, with application to computational cardiology’, founded by the European Union (Next Generation EU, Mission 4 Component 2). The present research is part of the activities of ‘Dipartimento di Eccellenza 2023–2027’, MUR, Italy, Dipartimento di Matematica, Politecnico di Milano. C.F. and C.P. have received funding from the European Union's Horizon 2020 under grant agreement No 952,166 REPAIR.

## Supporting information


Peer Review History

Supporting Information

The following datasets are provided as Supporting Information:
**Isovolumic and ejecting beat under baseline conditions: space statistics and representative points values (Fig. 2)**:
3D_model_isovolumic_beat_points.csv

3D_model_isovolumic_beat_stats.csv

3D_model_ejecting_beat_points.csv

3D_model_ejecting_beat_stats.csv

**3D model: isovolumic and ejecting beats for variable CaT (Fig. 4) and XB kinetics (Fig. 6)**:
3D_model_ejecting_beats_varying_CaT.csv

3D_model_ejecting_beats_varying_XBkinetics.csv

3D_model_isovolumic_beats_varying_CaT.csv

3D_model_isovolumic_beats_varying_XBkinetics.csv

**0D model: isometric and shortening twitches for variable CaT (Fig. 5) and XB kinetics (Fig. 7)**:
0D_model_isometric_twitch_varying_CaT.csv

0D_model_isometric_twitch_varying_XBkinetics.csv

0D_model_shortening_twitch_varying_CaT.csv

0D_model_shortening_twitch_varying_XBkinetics.csv

## Data Availability

This study does not involve any experimental data, as it is solely based on numerical simulations.
