# Supplementary material for: Elucidating the cellular determinants of the end‐systolic pressure‐volume relationship of the heart via computational modelling
Source: J Physiol. 2025 May 11;604(19):8006–27. doi: 10.1113/JP287282 (PMC13628554; doi:10.1113/JP287282)
Supplement: Supplementary file 1 [file TJP-604-8006-s007.pdf]

The Journal of Physiology

Elucidating the cellular determinants of the end-systolic pressure-volume relationship of the heart via computational modelling

Francesco Regazzoni, Corrado Poggesi, and Cecilia Ferrantini
DOI: 10.1113/JP287282

Corresponding author(s): Francesco Regazzoni (francesco.regazzoni@polimi.it)

The referees have opted to remain anonymous.

Review Timeline:

Submission Date:	01-Nov-2024
Editorial Decision:	02-Jan-2025
Revision Received:	01-Mar-2025
Accepted:	06-Apr-2025

Senior Editor: Natalia Trayanova

Reviewing Editor: Christian Vergara

Transaction Report:

(Note: With the exception of the correction of typographical or spelling errors that could be a source of ambiguity, letters and reports are not edited. Depending on transfer agreements, referee reports obtained elsewhere may or may not be included in this compilation. Referee reports are anonymous unless the Referee chooses to sign their reports.)

Dear Dr Regazzoni,

Re: JP-RP-2024-287282 "Elucidating the cellular determinants of the end-systolic pressure-volume relationship of the heart via computational modelling" by Francesco Regazzoni, Corrado Poggesi, and Cecilia Ferrantini

Thank you for submitting your manuscript to The Journal of Physiology. It has been assessed by a Reviewing Editor and by 2 expert referees and we are pleased to tell you that it is potentially acceptable for publication following satisfactory major revision.

Please advise your co-authors of this decision as soon as possible.

The referee reports are copied at the end of this email.

Please address all the points raised and incorporate all requested revisions or explain in your Response to Referees why a change has not been made. We hope you will find the comments helpful and that you will be able to return your revised manuscript within 2 months. If you require longer than this, please contact journal staff: jp@physoc.org. Please note that this letter does not constitute a guarantee for acceptance of your revised manuscript.

Your revised manuscript should be submitted online using the link in your Author Tasks: Link Not Available. This link is accessible via your account as Corresponding Author; it is not available to your co-authors. If this presents a problem, please contact journal staff (jp@physoc.org). Image files from the previous version are retained on the system. Please ensure you replace or remove any files that are being revised.

If you do not wish to submit a revised version of your manuscript, you must inform our journal staff (jp@physoc.org) or reply to this email to request withdrawal. Please note that a manuscript must be formally withdrawn from the peer review process at one journal before it may be submitted to another journal.

TRANSPARENT PEER REVIEW POLICY: To improve the transparency of its peer review process, The Journal of Physiology publishes online as supporting information the peer review history of all articles accepted for publication. Readers will have access to decision letters, including Editors' comments and referee reports, for each version of the manuscript, as well as any author responses to peer review comments. Referees can decide whether or not they wish to be named on the peer review history document.

ABSTRACT FIGURES: Authors are expected to use The Journal's premium BioRender account to create/redraw their Abstract Figures. Information on how to access this account is here:
https://physoc.onlinelibrary.wiley.com/journal/14697793/biorender-access.

This will enable Authors to create and download high-resolution figures. If authors have used the free BioRender service, they can use the instructions provided in the link above to download a high-resolution version suitable for publication.

The link provided should only be used for the purposes of this submission. Authors will be charged for figures created on this account if they are not related to this manuscript submission.

LANGUAGE EDITING AND SUPPORT FOR PUBLICATION: If you would like help with English language editing, or other article preparation support, Wiley Editing Services offers expert help, including English Language Editing, as well as translation, manuscript formatting, and figure formatting at www.wileyauthors.com/eeo/preparation. You can also find resources for Preparing Your Article for general guidance about writing and preparing your manuscript at www.wileyauthors.com/eeo/prepresources.

RIGOUR AND REPRODUCIBILITY: We recommend authors consult The Journal's Rigour and Reproducibility requirements to ensure that their manuscript is suitable for publication: https://physoc.onlinelibrary.wiley.com/pb-assets/hub-assets/physoc/documents/TJP-Rigour-and-Reproducibility-Requirements.pdf

REVISION CHECKLIST:

Check that your Methods section conforms to journal policy: https://jp.msubmit.net/cgi-bin/main.plex?form_type=display_requirements#methods.

Check that data presented conforms to the statistics policy: https://jp.msubmit.net/cgi-bin/main.plex?form_type=display_requirements#statistics.

Upload a full Response to Referees file. To create your 'Response to Referees': copy all the reports, including any comments from the Senior and Reviewing Editors, into a Microsoft Word, or similar, file and respond to each point, using font or background colour to distinguish comments and responses and upload as the required file type.

Please upload two versions of your manuscript text: one with all relevant changes highlighted and one clean version with no

changes tracked. The manuscript file should include all tables and figure legends, but each figure/graph should be uploaded as separate, high-resolution files.

You may also upload:

- 'Potential Cover Art' for consideration as the issue's cover image
- Appropriate Supporting Information (Video, audio or data set: see https://jp.msubmit.net/cgi-bin/main.plex?form_type=display_requirements#supp).

We look forward to receiving your revised submission.

If you have any queries, please reply to this email and we will be pleased to advise.

Yours sincerely,

Natalia Trayanova
Senior Editor
The Journal of Physiology

REQUIRED ITEMS

- Include a Key Points list in the article itself, before the Abstract.

- Author photo and profile. First or joint first authors are asked to provide a short biography (no more than 100 words for one author or 150 words in total for joint first authors) and a portrait photograph. These should be uploaded and clearly labelled together in a Word document with the revised version of the manuscript. See Information for Authors for further details.

- The reference list must be in alphabetical order, rather than numbered, to comply with our Journal format.

- Please upload separate high-quality figure files via the submission form.

- Please ensure that the Article File you upload is a Word file.

- Please include an Abstract Figure file, as well as the Figure Legend text within the main article file. The Abstract Figure is a piece of artwork designed to give readers an immediate understanding of the research and should summarise the main conclusions. If possible, the image should be easily 'readable' from left to right or top to bottom. It should show the physiological relevance of the manuscript so readers can assess the importance and content of its findings. Abstract Figures should not merely recapitulate other figures in the manuscript. Please try to keep the diagram as simple as possible and without superfluous information that may distract from the main conclusion(s). Abstract Figures must be provided by authors no later than the revised manuscript stage and should be uploaded as a separate file during online submission labelled as File Type 'Abstract Figure'. Please also ensure that you include the figure legend in the main article file. All Abstract Figures should be created using BioRender. Authors should use The Journal's premium BioRender account to export high-resolution images. Details on how to use and access the premium account are included as part of this email.

EDITOR COMMENTS

Reviewing Editor:

Methods Details:
See major remark n.3 by Referee 1

Comments to the Author (Required):

Dear Authors,

The Referees completed their review process. As you can see, Referee 1 raised some critical remarks. However, he did not reject the paper so that you have the possibility to properly answer them and, most importantly, to accordingly update the paper. Please put attention to the first major remark, i.e. the suitability of the journal.

Please also see 'Required Items' above.

REFEREE COMMENTS

Referee #1:

This research paper uses computational modelling to examine the end-systolic pressure-volume relationship of the heart. The authors investigate how this relationship, a key indicator of cardiac contractility, is affected by the mechanical mode of contraction (ejecting vs. isovolumetric). Their multiscale model incorporates subcellular mechanisms, allowing them to link cellular processes to organ-level function. The reviewers appreciated the work, nevertheless, the reviewers detected multiple critical issues which hinder consideration for publication.

Major issues:

- The paper does not adhere to the journal's policy for computational papers which states: "As in any experimental study, results from the model should add new scientific insights. The model should be based on physiological data where the parameters can be directly linked to experimental data. The model should be described in such a way that the results can be reproduced, i.e. all parameters are listed and any computation codes made available. Where possible there should be a detailed sensitivity analysis of the conclusions reached against the parameters proposed within the bounds of the data used to constrain them. Papers that develop integrative physiological models which link functions together or observations across scales would be encouraged." Each of these points necessitates individual resolution to align the manuscript with the journal's standards.

o The novel scientific insights are unclear from this paper. The authors assert that the ESPVR is not a single line. However, this assertion lacks novelty, as Otto Frank established this concept a century ago. (Line 506-507: "The model simulations presented in this paper indicate that this would result in an enhancement of the mechanical performance of ejecting beats, leading to a situation where the ESPV relationship of ejecting beats exceeds the isovolumetric curve." How is this different from the findings of Otto Frank?)

o The paper is not reproducible. The paper fails to provide sufficient detail regarding parameter choices and computational protocols. Many parameters are either unspecified or inadequately justified, rendering replication of the study unfeasible.

o The study does not link its computational results to specific experimental datasets. Given the availability of quantitative studies on pressure-volume loops in both animal and human subjects, such integration would strengthen the manuscript.

- The overall aim/objective of the paper remains ambiguous due to several inconsistencies:

o In lines 62-64, the authors reiterate Otto Frank's statement that the dependency of the ESPVR is primarily a property of cardiac muscle rather than the ventricular chamber. Subsequently, they proceed to develop a full 3D chamber model without explicitly justifying its necessity.

o Lines 73-76 discuss the difficulties in measuring variations in sarcomere length and the limitations of experimental studies. However, lines 77-79 contradict this by suggesting that such measurements are feasible.

o The final paragraph of the introduction makes several "for the first time" claims without adequately explaining the underlying need for the proposed 3D model.

o In lines 436-437, the discussion begins with the incorrect assumption that the field generally considers the ESPVR to be a single curve, contrary to Otto Frank's findings.

o In line 452, the authors mention contradictory findings in the literature but remain vague about their specifics. There is a lack of detailed summaries and references, leaving the manuscript's position relative to existing studies unclear.

o The introduction of "potential translation significance" in the last three paragraphs contrasts sharply with the rest of the manuscript, which does not address these aspects, further obfuscating the paper's primary aim.

- Methodological concerns on appropriateness of developed framework to expose chamber-level aspects of CaT and contractility interactions:

o The electrophysiological model only models the traveling wave and then assumes homogeneous and isotropic calcium dynamics across the entire domain, disregarding the heterogeneous and anisotropic nature of myocardial tissue. The impact of this assumption on the results is not addressed.

o The model lacks mechano-calcium feedback. The authors briefly mention that its inclusion would only have a quantitative, not qualitative, impact (lines 506-507). However, this claim is unsupported by any sensitivity analysis.

o It is unclear whether the Tor-ORd model has been validated or calibrated against mechanical data. Without such validation, the reliability of the one-way coupled results remains questionable.

- The comparison between the organ-scale and cell-scale model feels disconnected. The authors run the 3D organ model until a limit cycle, whilst they only seem to run the 0D cell model for one cycle. We advise the authors to substantially clarify the parallels and discrepancies between the two different scale simulations.

- Otto Frank originally highlighted the effect of insufficient ventricular emptying on subsequent ejection phases. The manuscript does not address how this factor is incorporated when running the model to a limit cycle.

Medium issues:

- In lines 40-42, the authors refer to a figure in Sagawa's work that reviewers could not locate using the provided DOI. Moreover, the Sagawa paper is not open access. This makes it difficult for the reader to the fundaments of this. We strongly advice the authors to seek permission to reproduce the relevant figure or create a schematic representation of the pressure-volume curves to aid reader comprehension.

- Referring to Fig. 1o as a "tissue scale model" is misleading, as the model only accounts for cell mechanics, as indicated by the constitutive relationship on line 202. More appropriate terminology would be "cell scale model".

- The manuscript does not elaborate on how the reference volume and constitutive parameters were chosen to ensure realistic EDPVRs. This selection critically influences the simulation outcomes and warrants detailed explanation.

- The manuscript does not account for different residual stresses within the tissue. How would these stresses affect the results.

- The connection between the prestressing parameters of the 3D model and those of the cell model is unclear and requires clarification.

- Figures 1a-e-f-j-k show heterogeneity in stretched sarcomere lengths resulting from the prestressing algorithm. The authors should discuss the realism of this heterogeneity and provide references to experimental evidence supporting stronger contractions in the apex versus the base.

Minor issue: The manuscript contains numerous self-citations, particularly concerning general constitutive laws, prestressing, and boundary condition choices. The journal's policy stipulates that self-citation should be minimised. The authors are encouraged to reference original sources where applicable to adhere to this guideline.

Referee #2:

This work explores the variability in the end-systolic pressure-volume (ESPV) relationship under different cellular conditions such as cross-bridge kinetics and calcium modulation. The authors show that the ESPV is not a curve, as usually thought, but rather an area. Such area can significantly differ from the ESPV obtained in isovolumetric experiments, especially with the case of increased or depressed cross-bridge kinetics and calcium transient. The effect is seen in both isolated myocyte and 3D multiscale simulations, confirming that the mechanism is intrinsic to sarcomere dynamics rather than other

macroscopic factors. The authors also provide a mechanistic insight, based on the temporal course of recruited units, which have some memory of the resting sarcomere length.

The manuscript is very well-written and well-structured, the hypotheses are clearly stated and the numerical experiments well-designed to support the thesis. This work is a perfect match for JP. Overall, there are no major issues and authors should be commended for the quality of this study. Some comments below should help to improve the manuscript.

1. The ESPVr is central to this study, but it is never clearly defined. I think this is intended by the authors, as they aims at showing that the ESPVr cannot be defined as a curve (and I like this approach). However, it may take a while to the reader to appreciate this. I would suggest to add a sketch in the intro to show the ESPV area (how would you define this in your experiments?) on top of the ESPV curve obtained from isovolumetric experiments. This should help to reconcile the figures in this manuscript and those we typically see in the literature (the ESPV is tangent to a series of PV loops).

2. Since the ESPV area in the experiments is not clearly shown, it is not clear how it relates to the ESPV lines (black curves in Figure 3 and on): do they overall, or the curve can be fully outside the area? (Seems so when Delta V_max or p_max are large.) Also, it seems you're only quantifying the distance of a PV loop from the black curve, rather than the width of the area itself, which is more qualitative.

3. In the introduction, paragraph 65-72, it is not clear to what extent cited studies performed similar experiments to this work. Did they provide similar results but only at single cell level and without providing any mechanistic insights? Or this results are known but never performed in 3D realistic settings?

4. Length-dependent effect, line 110: is the model accounting for this in a "natural" way, or is it imposed phenomenologically?

5. It seems you are not considering heterogeneity in APD spatial distribution in your electrophysiology model. Do you think that this limitation can have an impact on your results (3D simulations)? If the APD differs from point to point, then the temporal dynamics of force generation will differ. This won't probably change the results qualitatively, but it might be worth to mention.

6. Data availability: I think you should also answer the question on whether in silico data (and/or code) will be made available.

Minor:

1. The resolution of the figures is a bit low, small text is sometimes not very clear.

2. Line 344: "because to large an" ?

END OF COMMENTS

March 1st, 2025

The Journal of Physiology — Manuscript JP-RP-2024-287282
“Elucidating the cellular determinants of the end-systolic pressure-volume relationship of the heart via computational modelling”

Answer to Referees

Dear Editor,

Thank you for your feedback on the manuscript. We would like to thank the Referees for their suggestions and constructive criticism. All of their comments have been carefully considered, and the manuscript has been modified accordingly.

Please, find below the detailed answers. Modifications to the paper with respect to the original submission have been highlighted in blue color, both in the manuscript and in the present letter.

Thank you for your time and your consideration.

Best regards,

Francesco Regazzoni, Corrado Poggesi, Cecilia Ferrantini

EDITOR COMMENTS

Reviewing Editor:

Methods Details:

See major remark n.3 by Referee 1

Comments to the Author (Required):

Dear Authors,

The Referees completed their review process. As you can see, Referee 1 raised some critical remarks. However, he did not reject the paper so that you have the possibility to properly answer them and, most importantly, to accordingly update the paper. Please put attention to the first major remark, i.e. the suitability of the journal.

Please also see 'Required Items' above.

We sincerely thank the Reviewing Editor for the opportunity to address the Referees' comments. We appreciate the constructive feedback, which has helped us improve the clarity and impact of our manuscript.

We have carefully addressed the concerns raised by Referee 1, particularly regarding methodological details. Specifically, we have made explicit the definition of the ESPV relationship, clarified the novelty of our study, and enhanced reproducibility by explicitly reporting all parameter values and providing the full in silico dataset as supplementary material. Additionally, we have improved the discussion of translational significance and refined the rationale for our modeling choices.

See below for detailed responses to each comment.

REFEREE COMMENTS

Referee #1:

This research paper uses computational modelling to examine the end-systolic pressure-volume relationship of the heart. The authors investigate how this relationship, a key indicator of cardiac contractility, is affected by the mechanical mode of contraction (ejecting vs. isovolumetric). Their multiscale model incorporates subcellular mechanisms, allowing them to link cellular processes to organ-level function. The reviewers appreciated the work, nevertheless, the reviewers detected multiple critical issues which hinder consideration for publication.

Major issues:

- **The paper does not adhere to the journal's policy for computational papers which states: "As in any experimental study, results from the model should add new scientific insights. The model should be based on physiological data where the parameters can be directly linked to experimental data. The model should be described in such a way that the results can be reproduced, i.e. all parameters are listed and any computation codes made available. Where possible there should be a detailed sensitivity analysis of the conclusions reached against the parameters proposed within the bounds of the data used to constrain them. Papers that develop integrative physiological models which link functions together or observations across scales would be encouraged." Each of these points necessitates individual resolution to align the manuscript with the journal's standards.**

    - **The novel scientific insights are unclear from this paper. The authors assert that the ESPVR is not a single line. However, this assertion lacks novelty, as Otto Frank established this concept a century ago. (Line 506-507: "The model simulations presented in this paper indicate that this would result in an enhancement of the mechanical performance of ejecting beats, leading to a situation where the ESPV relationship of ejecting beats exceeds the isovolumetric curve." How is this different from the findings of Otto Frank?)**

      With regard to ejecting beats, we have shown that ESPV points do not align along a single curve but instead form a scattered area. Frank compared isovolumetric and ejecting beats but never demonstrated that ejecting beats could exceed the isovolumetric ESPV curve. This evidence was provided nearly a century later by Little, whose experiments revealed not only the well-established ejection-dependent deactivation but also ejection-dependent activation. While Frank observed only the deactivating effect of shortening, our study shows that both activating and deactivating effects are possible, depending on subcellular kinetics. Furthermore, through this computational investigation, we have identified the molecular mechanisms that determine the positioning of ESPV points within this area.

      To avoid potential misunderstandings, we have provided in the Introduction a clear and unambiguous definition of the research questions addressed in this study, while also summarising previous findings:

Given the lack of consensus on the nature of the ESPV relationship, it is useful to establish clear definitions to unambiguously frame the issues under investigation. A commonly used operational definition of ESPV involves constructing the envelope of multiple PV loops, as depicted in red in Figure 1a. Specifically, for a given end-diastolic volume (EDV), varying the afterload resistance generates a family of PV loops, all of which are tangent to a single curve. This curve defines the ejecting ESPV relationship associated with that EDV. Conversely, if we consider beats under isovolumetric conditions (i.e., when the aortic valve remains closed), we obtain the isovolumetric ESPV curve by connecting the peak pressure-volume points recorded at different volumes, as shown in black in Figure 1b.

Now, let us return to the ejecting ESPV relationship and imagine repeating this procedure while varying EDV. According to the view that the ESPV relationship is unique (e.g., as proposed by Suga, Sagawa, and Shoukas 1973), the PV loops at different EDVs will all be tangent to a single, well-defined curve, as illustrated in Figure 1c (left), which depends solely on cardiac inotropism. An alternative hypothesis, supported e.g. by Han et al. 2019, is that the ESPV relationships at different EDVs define a broader region rather than a single curve — referred to as the ESPV zone, as shown in Figure 1c (right).

A second key question is how the ejecting ESPV relationship (whether a curve or a zone) compares to the isovolumetric ESPV curve. As illustrated in Figure 1d, three possible scenarios can be considered: (i) shortening activation, where ejection enhances contractile force, causing PV loops to exceed the isovolumetric ESPV curve as shown for example by Igarashi, Cheng, and Little 1991; (ii) shortening deactivation, where ejection reduces contractility, causing PV loops to fall below the isovolumetric ESPV curve as originally described by Frank 1899; or (iii) no effect of shortening, in which case the ejecting ESPV relationship essentially coincides with the isovolumetric ESPV curve as shown by Suga, Sagawa, and Shoukas 1973.

It is important to note that the above two points are independent. The first concerns the fundamental nature of the ejecting ESPV relationship — whether it is a well-defined curve or a broader region in the PV plane. The second question addresses the physiological effects of ejection on contractility — whether ejection enhances, reduces, or has no impact on the ESPV relationship.

- **The paper is not reproducible. The paper fails to provide sufficient detail regarding parameter choices and computational protocols. Many parameters are either unspecified or inadequately justified, rendering replication of the study unfeasible.**

  We thank the Referee for highlighting this important issue. We have now provided the full set of parameter values used in the simulations and clarified the criteria for their

selection. Additionally, we have now provided the full dataset of in silico data generated and analysed in this study as supplementary material.

- **The study does not link its computational results to specific experimental datasets. Given the availability of quantitative studies on pressure-volume loops in both animal and human subjects, such integration would strengthen the manuscript.**

  Clinical acquisition of pressure-volume (PV) loops in healthy individuals is uncommon, making datasets of human control subjects scarce compared to data from patients with cardiovascular disease. Still, the PV loops predicted by our model under baseline conditions fall within the range of values reported in the literature for healthy subjects (Bastos et al. 2020; Seemann et al. 2019; Kawel-Boehm et al. 2020), as we now explicitly state in the Methods section. Clinical pressure-volume (PV) loops acquisition is typically performed using a 4–7 F catheter with 12 electrodes spaced evenly, and a solid-state pressure sensor at the centre (Bastos et al. 2020). The catheter is inserted via either the trans-aortic or trans-septal approach, with the tip positioned in the left ventricle (LV) apex, and the most proximal electrode placed above the aortic or mitral valve, depending on the approach. Bastos and colleagues reached conclusions similar to those of a number of similar studies conducted with different methodologies (e.g. Seemann et al. 2019; Kawel-Boehm et al. 2020; Argirò et al. 2022) and it was highlighted that the End-Systolic Pressure-Volume Relationship (ESPVR) elastance increases with positive inotropic interventions and diseases such as HCM, while it decreases with negative inotropic interventions or myocardial damage in HFrEF.

  That said, the goal of this study is neither to conduct a patient-specific analysis nor to replicate an experimental procedure *in silico*. Rather, the model, once calibrated under baseline conditions, serves as a tool to systematically investigate the effects of targeted changes in cellular variables — specifically, crossbridge (XB) kinetics and calcium transient (CaT) duration — on the PV loop as end-diastolic volume (EDV) and afterload resistance vary. For these analyses, direct comparison with experimental data is not feasible. While XB kinetics and CaT duration can be influenced by pharmacological interventions or disease, such changes never occur in isolation in vivo. The strength of the computational approach lies precisely in its ability to isolate and quantify the contributions of these individual factors to cardiac function, which would be challenging, if not impossible, to achieve experimentally.

- **The overall aim/objective of the paper remains ambiguous due to several inconsistencies:**

  - **In lines 62-64, the authors reiterate Otto Frank's statement that the dependency of the ESPVR is primarily a property of cardiac muscle rather than the ventricular chamber. Subsequently, they proceed to develop a full 3D chamber model without explicitly justifying its necessity.**

    We thank the Referee for pointing out that the rationale for using both tissue-level and chamber-level simulations was not clearly presented in the first version of the manuscript. The use of both models is intended to investigate the extent to which the results can be considered as intrinsic properties of the tissue or as organ-level

phenomena, bacause Otto Frank's statement is still debated (see e.g. Han et al. 2019; 2021). We have now added the following sentence at the beginning of the "Methods" section:

We considered an *in silico* model of human cardiac electromechanics, based on the iHEART Simulator (F. Regazzoni et al. 2022)~~Fedele et al. 2023),~~ which incorporates a biophysically detailed representation of electrophysiological and mechanical processes, along with their coupling. We considered two simulation settings: tissue-level simulations, which ignore 3D effects, and chamber-level simulations. Our aim was to analyse the impact of shortening on contractility in both settings and to determine to what extent the observed effects are due to intrinsic tissue properties or arise as organ-level phenomena.

Furthermore, we have motivated the use of a 3D model in the "Introduction" section:

Disease-associated variations in XB kinetics and calcium transient (CaT) profile are hallmarks of heart failure with both reduced and preserved ejection fraction (e.g. hypertrophic cardiomyopathy). In addition, the relative expression of alpha and beta myosin (severely affecting XB kinetics) in human myocardium can vary in life, even in physiological settings. This reinforces the need of a comprehensive mathematical model that includes the cellular and 3D organ level to predict the effects of these changes in XB cycling and CaT profile.

- **Lines 73-76 discuss the difficulties in measuring variations in sarcomere length and the limitations of experimental studies. However, lines 77-79 contradict this by suggesting that such measurements are feasible.**

  Lines 73–76 highlight the practical challenges of measuring changes in sarcomere length (SL) variation, particularly under physiological conditions, but do not imply that such measurements are entirely unfeasible. The work mentioned in lines 77–79 is, to the best of our knowledge, the only available data on this topic. However, with only one set of measurements available, it is clear that much remains unknown. We have added the following information for clarity:

These authors found broad distributions for both diastolic SL (1.8-2.4) and systolic SL (1.3-2.1 μm), providing valuable insights despite the limitations of extrapolating these findings to human physiology.

- **The final paragraph of the introduction makes several "for the first time" claims without adequately explaining the underlying need for the proposed 3D model.**

Thank you for this suggestion, we have amended the text accordingly. The rationale for using a 3D model has also been also better highlighted as discussed in the answer to the previous questions.

- **In lines 436-437, the discussion begins with the incorrect assumption that the field generally considers the ESPVR to be a single curve, contrary to Otto Frank's findings.**

While Frank's diagrams were influential in German cardiac physiology, they were largely unknown abroad until 1969, when Hiroyuki Suga began to present similar approaches for warm-blooded animals as Frank had done for the frog. Suga showed that with a constant contractile state and afterload resistance, a progressive reduction in ventricular filling pressure causes the loops to shift towards lower volumes at both end systole and end diastole. Connecting the resulting end-systolic pressure-volume points gives a reasonably linear ESPV relationship is obtained. The linear ESPVR is characterised by a slope (E_{es}) and a volume axis intercept (V_0). In contrast, the diastolic pressure-volume points define a nonlinear end-diastolic pressure-volume relationship (EDPVR). As the afterload resistance is increased with a constant preload pressure, the loops become narrower and longer and, under idealised conditions, the end-systolic pressure-volume points fall on the same ESPV relationship as obtained with preload reduction.

As discussed in the answer to the first question, we have modified the Introduction section to better clarify this aspect. Moreover, at the beginning of the Discussion section, we have summarised previous work in the literature supporting the view of the ESPV relationship as a zone instead of a single line:

Instead, this study suggests that, by connecting the end-systolic points obtained by varying the preload and afterload resistances, we do not obtain a single curve, but rather a region within the pressure-volume plane. This is in line with force-length recordings on intact trabeculae by De Tombe and Little 1994 and Han et al. 2019, as well as previous numerical studies of both single cells (Landesberg 1996; Shimizu, Todaka, and Burkhoff 2002; Iribe et al. 2014; Kalda, Peterson, and Vendelin 2015) and simplified 3D models (Pironet et al. 2013).

- **In line 452, the authors mention contradictory findings in the literature but remain vague about their specifics. There is a lack of detailed summaries and references, leaving the manuscript's position relative to existing studies unclear.**

As discussed earlier, we have now made the terms of the debate unambiguous starting with the Introduction. In addition, we have recalled some references in the Discussion:

[...] thus reconciling the apparently contradictory findings in the literature, such as those of Frank 1899 and De Tombe and Little 1994.

- **The introduction of "potential translation significance" in the last three paragraphs contrasts sharply with the rest of the manuscript, which does not address these aspects, further obfuscating the paper's primary aim.**

  In the Introduction we have introduced a paragraph explaining the clinical rationale for this study:

Disease-associated variations in XB kinetics and calcium transient (CaT) profile are hallmarks of heart failure with both reduced and preserved ejection fraction (e.g. hypertrophic cardiomyopathy). In addition, the relative expression of alpha and beta myosin (severely affecting XB kinetics) in human myocardium can vary in life, even in physiological settings. This reinforces the need of a comprehensive mathematical model that includes the cellular and 3D organ level to predict the effects of these changes in XB cycling and CaT profile.

  In addition, in the Discussion we have anticipated the paragraph on the limitations of the study, to make it clear that the translational significance will only be addressed in the remaining two paragraphs. We have also explicitly stated that clinical translational aspects were not directly investigated in this study:

We now address the potential translational significance of this work, although these aspects were not specifically investigated in this study.

- **Methodological concerns on appropriateness of developed framework to expose chamber-level aspects of CaT and contractility interactions:**

  - **The electrophysiological model only models the traveling wave and then assumes homogeneous and isotropic calcium dynamics across the entire domain, disregarding the heterogeneous and anisotropic nature of myocardial tissue. The impact of this assumption on the results is not addressed.**

    The electrical conductivity tensor in the eikonal-diffusion model explicitly accounts for local fibre directions, thus ensuring anisotropy. To clarify this point, we have added the following sentence:

The eikonal-diffusion model incorporates an anisotropic conductivity tensor that reflects the local fibre and sheetlet orientations.

With regard to heterogeneity, we have now acknowledged this as a limitation of the study and have discussed why its impact is expected to be negligible, based on the results of previous work:

In addition, to limit the computational cost, we used the Eikonal equation to model action potential propagation, neglecting mechano-electrical feedback and heterogeneities in action potential duration. However, previous studies have shown that, as long as the overall electrophysiological activity remains within physiological ranges, these modelling simplifications have a negligible impact on the pressure-volume loop (Salvador et al. 2022; Zappon et al. 2024).

- **The model lacks mechano-calcium feedback. The authors briefly mention that its inclusion would only have a quantitative, not qualitative, impact (lines 506-507). However, this claim is unsupported by any sensitivity analysis.**

  The cellular mechanisms investigated in this paper are independent of the mechano-calcium feedback, which would only add up, potentially shifting PV loops but without being able to reverse the trend. This is why we stated that the inclusion of mechano-calcium feedback would have had no impact at the qualitative level.

  In addition, we are finalising a manuscript looking at the effects of mechano-calcium feedback on overall mechanical cardiac function, which shows that the impact of mechano-calcium feedback on the PV loop is very small, and in any case negligible compared to the variations studied in this paper. Therefore, we are confident that the results of this study would not change significantly, even on a quantitative level.

- **It is unclear whether the Tor-ORd model has been validated or calibrated against mechanical data. Without such validation, the reliability of the one-way coupled results remains questionable.**

  In this study the ToR-ORd model is used to simulate the calcium transient. As mentioned in the manuscript, the model has been calibrated and extensively validated in its original reference (Tomek et al. 2019). The ToR-ORd model is then coupled unidirectionally with the RDQ20-MF model. The resulting mechanical output has been calibrated and validated against human data, as described in detail in Francesco Regazzoni, Dedè, and Quarteroni 2020, where calcium dynamics are indeed simulated by the ToR-ORd model. Notably, the same one-way coupling approach is used in this study.

- **The comparison between the organ-scale and cell-scale model feels disconnected. The authors run the 3D organ model until a limit cycle, whilst they only seem to run the 0D cell**

model for one cycle. We advise the authors to substantially clarify the parallels and discrepancies between the two different scale simulations.

We appreciate the Referee's suggestion and have better clarified this point in the revised manuscript. Specifically, the electrophysiology component of the 0D cell model is run for multiple cycles until a limit cycle is reached, consistent with the approach used for the 3D organ model. However, the remainder of the simulation — the active force generation coupled with the viscoelastic components — is only run for a single cycle. This is because running additional cycles would give identical results, as the system is already in a perfect steady state. We have made this point explicit in the Methods section, with the following clarification:

> We note that, unlike in the left ventricle simulations, it is not necessary to run multiple cycles to allow the transient effects of the initial conditions to decay. This is because the simplicity of this model ensures that the initial condition is already in a perfect steady state.

- **Otto Frank originally highlighted the effect of insufficient ventricular emptying on subsequent ejection phases. The manuscript does not address how this factor is incorporated when running the model to a limit cycle.**

In this work, the LV model is not coupled to a closed-loop circulatory model, so it is not affected by feedback mechanisms such as those mentioned by the Referee. This modelling approach was specifically chosen to eliminate such effects, allowing full control over preload and afterload and enabling a direct assessment of their impact. The model is designed to maintain a fixed EDV level. As this was not clearly explained, we have added the following description to the manuscript:

> Instead, during ejecting beats the valves were kept closed until a pressure of 80 mmHg was reached, and then the LV model was coupled to a two-element Windkessel afterload model as described in Stella et al. 2022 (see Figure 2a). A second isovolumetric phase was simulated at the end of ejection i.e., when the volume time derivative reached zero. Finally, in the last phase, a ramp was applied to return the volume to its end-diastolic value. We deliberately chose this modelling approach over more complex closed-loop (F. Regazzoni et al. 2022; Hirschvogel et al. 2017) in order to maintain full control over preload and afterload, allowing us to assess their effects directly.

We note that the aim of this paper is not to reproduce Otto Frank's experiments in silico but rather — inspired by his observations and subsequent studies — to use the computational model as a tool for mechanistically investigating the effects of cellular mechanisms at the organ scale.

Medium issues:

- **In lines 40-42, the authors refer to a figure in Sagawa's work that reviewers could not locate using the provided DOI. Moreover, the Sagawa paper is not open access. This makes it difficult for the reader to the fundaments of this. We strongly advice the authors to seek permission to reproduce the relevant figure or create a schematic representation of the pressure-volume curves to aid reader comprehension.**

  The figure originally redrawn by Sagawa is reproduced in textbooks as well as in recent, more easily accessible reviews of Sagawa's original work, such as (Kuhtz-Buschbeck et al. 2018). In addition to Sagawa's original work, the aforementioned review is now cited in the revised text, which is open access and therefore easily accessible.

  We have doublechecked the DOI of Sagawa's work, and we confirm that it is correct.

- **Referring to Fig. 1o as a "tissue scale model" is misleading, as the model only accounts for cell mechanics, as indicated by the constitutive relationship on line 202. More appropriate terminology would be "cell scale model".**

  Following the Referee's suggestion we now refer to the 0D model as "cell scale model".

- **The manuscript does not elaborate on how the reference volume and constitutive parameters were chosen to ensure realistic EDPVRs. This selection critically influences the simulation outcomes and warrants detailed explanation.**

  To recover of the reference configuration, we used a pressure value from a previous work based on the same geometry, as now specified in the revised manuscript:

  > [...] we recovered the unstressed reference configuration ~~using the inverse displacement algorithm(Barnafi, Regazzoni, and Riccobelli 2024)~~, assuming a pre-stressing LV pressure of 7.5 mmHg as in F. Regazzoni et al. 2022.

  Instead, the material stiffness was tuned together with the Windkessel parameters and active contractility to obtain a realistic pressure-volume loop, as now detailed in the manuscript:

  > The parameters used in this study are reported in Table 2. To select their values, we first focused on electrophysiology by setting the conductivity coefficients used in Fedele et al. 2023 and then selecting the parameter c_0 that guarantees a physiological conduction velocity along the fibre direction (Augustin et al. 2021). Next, we manually tuned the passive stiffness of the material, the XB stiffness and the Windkessel model parameters (afterload resistance R_{ar} and compliance C_{ar}) to have a pressure-volume loop within physiological ranges (Bastos et al. 2020; Seemann et al. 2019; Kawel-Boehm et al. 2020; Argirò et al. 2022).

- **The manuscript does not account for different residual stresses within the tissue. How would these stresses affect the results.**

  We have addressed this limitation in the Discussion section, where we acknowledge the assumption of the existence of a reference unstressed configuration, which is standard in many cardiac computational models:

  > Another limitation is that we have ignored residual stress and assumed the existence of a reference unstressed configuration, as is common in cardiac computational models (Peirlinck et al. 2018; Gerach et al. 2021; Marx et al. 2022).

- **The connection between the prestressing parameters of the 3D model and those of the cell model is unclear and requires clarification.**

  It is not clear to us whether the Referee is referring to the reference configuration recovery procedure or rather to the applied initial condition (preload). The reference configuration recovery is only relevant to the 3D model, as the 0D case does not involve a geometry that requires a stress-free configuration calculation. On the other hand, for the preload setting, we have now specified more precisely how the $\bar{T}$ parameter is determined:

  > where $\bar{T}$ ~~is a constant that depends on the above parameters~~ is set equal to threshold $T_{th} + k_s \lambda_{ED}$, with λ_{ED} being the end-diastolic strain at hand. This choice ensures that at the switch from the isometric to the shortening phase the forces are in equilibrium

  The link between 3D and 0D preload is ensured by selecting the spatially averaged end-diastolic SL observed in the 3D simulation in the latter, as explained in the next sentence:

  > Shortening twitches were performed by considering three values of SL_{ED} (2.0779, 2.1783, 2.2765 μM, chosen by taking the mean end-diastolic SL in the LV tests reported in Figure 2),

- **Figures 1a-e-f-j-k show heterogeneity in stretched sarcomere lengths resulting from the prestressing algorithm. The authors should discuss the realism of this heterogeneity and provide references to experimental evidence supporting stronger contractions in the apex versus the base.**

> As discussed in the introduction, experimental data on the spatial distribution of sarcomere length (SL) during the heartbeat are very limited. A technique to measure SL in living cardiac tissue was presented by Botcherby et al. 2013. This method allows SL imaging in Langendorff-perfused rodent hearts. However, this technique was not performed in situ, with all pericardial and thoracic constraints preserved. To our knowledge, the only direct in situ measurement of SL in beating hearts has been provided by the Fukuda's laboratory (Kobirumaki-Shimozawa et al. 2016). They developed a high speed (100 frames per second), high resolution (20 nm) imaging system for myocardial sarcomeres in living mice. This system was used to perform three-dimensional analysis of sarcomere dynamics in left ventricular myocytes during the cardiac cycle, simultaneously with electrocardiogram and left ventricular pressure measurements. The working range of SL was very wide, with a diastolic range of 1.8-2.4 and systolic range of 1.3-2.1 μm, with significant space variability within the LV, although a comprehensive characterization within specific regions (e.g. apex vs base) was not addressed.

Minor issue:

The manuscript contains numerous self-citations, particularly concerning general constitutive laws, prestressing, and boundary condition choices. The journal's policy stipulates that self-citation should be minimised. The authors are encouraged to reference original sources where applicable to adhere to this guideline.

Following the Referee's suggestion, we have carefully reviewed the self-citations throughout the manuscript. We have removed the reference to Fedele et al. 2023 where the iHEART model is first mentioned, leaving only the reference to F. Regazzoni et al. 2022. In addition, we have removed the reference to Barnafi, Regazzoni, and Riccobelli 2024 when discussing the reference configuration recovery algorithm, as the specification of the algorithm used is not essential for understanding the manuscript. Furthermore, in the description of the active force generation model, we have rephrased the following sentence as follows, and removed the citation to Francesco Regazzoni, Dedè, and Quarteroni 2018:

> Although the explicit description of end-to-end interactions typically leads to prohibitive computational costs (Rice and de Tombe 2004), ~~thanks to the strategy proposed in (Francesco Regazzoni, Dedè, and Quarteroni 2018),~~ the model is formulated as a system of ordinary differential equations, by introducing suitable closure relationships.

We carefully examined the remaining self-citations, particularly in the description of the *in silico* model. We found that each of the remaining self-citations refers to a model or numerical method originally introduced in the cited paper. These references are necessary to ensure the completeness of the manuscript and to allow the reader to trace the origin of the methods used. We believe that this practice is in line with the policy of the Journal and ensures transparency and clarity for the reader.

Referee #2:

This work explores the variability in the end-systolic pressure-volume (ESPV) relationship under different cellular conditions such as cross-bridge kinetics and calcium modulation. The authors show that the ESPV is not a curve, as usually thought, but rather an area. Such area can significantly differ from the ESPV obtained in isovolumetric experiments, especially with the case of increased or depressed cross-bridge kinetics and calcium transient. The effect is seen in both isolated myocyte and 3D multiscale simulations, confirming that the mechanism is intrinsic to sarcomere dynamics rather than other macroscopic factors. The authors also provide a mechanistic insight, based on the temporal course of recruited units, which have some memory of the resting sarcomere length.

The manuscript is very well-written and well-structured, the hypotheses are clearly stated and the numerical experiments well-designed to support the thesis. This work is a perfect match for JP. Overall, there are no major issues and authors should be commended for the quality of this study. Some comments below should help to improve the manuscript.

- **The ESPVr is central to this study, but it is never clearly defined. I think this is intended by the authors, as they aims at showing that the ESPVr cannot be defined as a curve (and I like this approach). However, it may take a while to the reader to appreciate this. I would suggest to add a sketch in the intro to show the ESPV area (how would you define this in your experiments?) on top of the ESPV curve obtained from isovolumetric experiments. This should help to reconcile the figures in this manuscript and those we typically see in the literature (the ESPV is tangent to a series of PV loops).**

  Following the Referee's suggestion, we have introduced a new figure (Figure 1 in the revised manuscript) to illustrate these definitions more clearly. Specifically, we now provide a precise definition of the ejecting and isovolumetric ESPV relationships, as well as the concepts of the ESPV curve versus the ESPV zone. Furthermore, we explicitly define the notions of shortening activation and shortening deactivation to frame the discussion on how ejection influences contractility. We have added these paragraphs to the Introduction:

> Given the lack of consensus on the nature of the ESPV relationship, it is useful to establish clear definitions to unambiguously frame the issues under investigation. A commonly used operational definition of ESPV involves constructing the envelope of multiple PV loops, as depicted in red in Figure 1a. Specifically, for a given end-diastolic volume (EDV), varying the afterload resistance generates a family of PV loops, all of which are tangent to a single curve. This curve defines the ejecting ESPV relationship associated with that EDV. Conversely, if we consider beats under isovolumetric conditions (i.e., when the aortic valve remains closed), we obtain the isovolumetric ESPV curve by connecting the peak pressure-volume points recorded at different volumes, as shown in black in Figure 1b.
>
> Now, let us return to the ejecting ESPV relationship and imagine repeating this procedure while varying EDV. According to the view that the ESPV relationship

is unique (e.g., as proposed by Suga, Sagawa, and Shoukas 1973), the PV loops at different EDVs will all be tangent to a single, well-defined curve, as illustrated in Figure 1c (left), which depends solely on cardiac inotropism. An alternative hypothesis, supported e.g. by Han et al. 2019, is that the ESPV relationships at different EDVs define a broader region rather than a single curve — referred to as the ESPV zone, as shown in Figure 1c (right).

A second key question is how the ejecting ESPV relationship (whether a curve or a zone) compares to the isovolumetric ESPV curve. As illustrated in Figure 1d, three possible scenarios can be considered: (i) shortening activation, where ejection enhances contractile force, causing PV loops to exceed the isovolumetric ESPV curve as shown for example by Igarashi, Cheng, and Little 1991; (ii) shortening deactivation, where ejection reduces contractility, causing PV loops to fall below the isovolumetric ESPV curve as originally described by Frank 1899; or (iii) no effect of shortening, in which case the ejecting ESPV relationship essentially coincides with the isovolumetric ESPV curve as shown by Suga, Sagawa, and Shoukas 1973.

It is important to note that the above two points are independent. The first concerns the fundamental nature of the ejecting ESPV relationship — whether it is a well-defined curve or a broader region in the PV plane. The second question addresses the physiological effects of ejection on contractility — whether ejection enhances, reduces, or has no impact on the ESPV relationship.

These additions should help reconcile our findings with the conventional representation of ESPV curves in the literature, making the manuscript more accessible to readers. We appreciate the Referee's valuable suggestion, which has contributed to a clearer presentation of key concepts of this study.

- **Since the ESPV area in the experiments is not clearly shown, it is not clear how it relates to the ESPV lines (black curves in Figure 3 and on): do they overall, or the curve can be fully outside the area? (Seems so when Delta V_max or p_max are large.) Also, it seems you're only quantifying the distance of a PV loop from the black curve, rather than the width of the area itself, which is more qualitative.**

  We thank the Referee for this insightful comment: as we now clarified in the Introduction, the two research questions we address are distinct: one concerns the nature of the *ejecting ESPV relationship* (whether it is best described as a curve or a zone), while the other examines *ejecting vs isovolumetric ESPV* relationships (i.e., whether shortening has a positive, negative, or negligible effect on activation).

  In Figure 3 and subsequent figures, we quantitatively assess the second aspect (the effect of ejection) using the indices ΔV_{80}, $\Delta V_{\max}$ and $\Delta p_{\max}$. Conversely, we approach the first aspect (curve vs zone) qualitatively, as a rigorous construction of the ESPV zone would require a

substantially larger number of PV loops. Additionally, its boundaries would be influenced by the specific EDV considered.

- **In the introduction, paragraph 65-72, it is not clear to what extent cited studies performed similar experiments to this work. Did they provide similar results but only at single cell level and without providing any mechanistic insights? Or this results are known but never performed in 3D realistic settings?**

  The cited papers reported results consistent with those of the present study, confirming that the ESPV relationship is load-dependent. However, in these studies, the cellular mechanisms underlying this behaviour were not clearly dissected, and a realistic 3D setting was never considered.
  We have highlighted the relationship with these results in the Discussion:

> Instead, this study suggests that, by connecting the end-systolic points obtained by varying the preload and afterload resistances, we do not obtain a single curve, but rather, a region within the pressure-volume plane. This is in line with force-length recordings on intact trabeculae by De Tombe and Little 1994 and Han et al. 2019, as well as previous numerical studies of both single cells (Landesberg 1996; Shimizu, Todaka, and Burkhoff 2002; Iribe et al. 2014; Kalda, Peterson, and Vendelin 2015) and simplified 3D models (Pironet et al. 2013).

- **Length-dependent effect, line 110: is the model accounting for this in a "natural" way, or is it imposed phenomenologically?**

  In the model the calcium-sensitivity of troponin features has phenomenological dependence on SL. We have now stated it more clearly:

> The model also accounts for length-dependent effects on the thin-filament activation, through an effective calcium-sensitivity of troponin that increases with the SL according to a prescribed linear relationship, [...]

- **It seems you are not considering heterogeneity in APD spatial distribution in your electrophysiology model. Do you think that this limitation can have an impact on your results (3D simulations)? If the APD differs from point to point, then the temporal dynamics of force generation will differ. This won't probably change the results qualitatively, but it might be worth to mention.**

  Thank you for bringing this important point to our attention. We have added the following sentence in Discussion section.

In addition, to limit the computational cost, we have used the Eikonal equation to model the action potential propagation, neglecting mechano-electrical feedback and heterogeneities in the action potential duration. However, previous studies have shown that, that as long as the overall electrophysiological activity remains within physiological ranges, these modelling simplifications have a negligible impact on the pressure-volume loop (Salvador et al. 2022; Zappon et al. 2024).

- **Data availability: I think you should also answer the question on whether in silico data (and/or code) will be made available.**

  The finite element code cannot be made available due to the policy of the library life^x. However, in the revised manuscript, we have included tables listing the parameters used to ensure, in principle, reproducibility in other codes. Additionally, we have now provided the full dataset of in silico data produced and analysed in this study as supplementary material.

Minor:

- **The resolution of the figures is a bit low, small text is sometimes not very clear.**

  We apologize for the inconvenience, which was likely caused by the file's conversion to PDF. Full-resolution figures are now provided to the journal through the submission system.

- **Line 344: "because to large an" ?**

  Fixed, thank you.

References

Argirò, Alessia, Hannah Rosenblum, Jan Griffin, Jaya Batra, Francesco Cappelli, Daniel Burkhoff, Mathew Maurer, and Iacopo Olivotto. 2022. 'Sex Related Differences in Exercise Performance in Patients with Hypertrophic Cardiomyopathy: Hemodynamic Insights through Non-Invasive Pressure Volume Analysis'. *International Journal of Cardiology* 351 (March):78–83. https://doi.org/10.1016/j.ijcard.2021.12.045.

Augustin, Christoph M., Matthias A.F. Gsell, Elias Karabelas, Erik Willemen, Frits W. Prinzen, Joost Lumens, Edward J. Vigmond, and Gernot Plank. 2021. 'A Computationally Efficient Physiologically Comprehensive 3D–0D Closed-Loop Model of the Heart and Circulation'. *Computer Methods in Applied Mechanics and Engineering* 386 (December):114092. https://doi.org/10.1016/j.cma.2021.114092.

Barnafi, N.A., F. Regazzoni, and D. Riccobelli. 2024. ‘Reconstructing Relaxed Configurations in Elastic Bodies: Mathematical Formulations and Numerical Methods for Cardiac Modeling’. *Computer Methods in Applied Mechanics and Engineering* 423 (April):116845. https://doi.org/10.1016/j.cma.2024.116845.

Bastos, Marcelo B, Daniel Burkhoff, Jiri Maly, Joost Daemen, Corstiaan A den Uil, Koen Ameloot, Mattie Lenzen, et al. 2020. ‘Invasive Left Ventricle Pressure–Volume Analysis: Overview and Practical Clinical Implications’. *European Heart Journal* 41 (12): 1286–97. https://doi.org/10.1093/eurheartj/ehz552.

De Tombe, P. P., and W. C. Little. 1994. ‘Inotropic Effects of Ejection Are Myocardial Properties’. *American Journal of Physiology-Heart and Circulatory Physiology* 266 (3): H1202–13. https://doi.org/10.1152/ajpheart.1994.266.3.H1202.

Fedele, Marco, Roberto Piersanti, Francesco Regazzoni, Matteo Salvador, Pasquale Claudio Africa, Michele Bucelli, Alberto Zingaro, Luca Dede’, and Alfio Quarteroni. 2023. ‘A Comprehensive and Biophysically Detailed Computational Model of the Whole Human Heart Electromechanics’. *Computer Methods in Applied Mechanics and Engineering* 410 (May):115983. https://doi.org/10.1016/j.cma.2023.115983.

Gerach, Tobias, Steffen Schuler, Jonathan Fröhlich, Laura Lindner, Ekaterina Kovacheva, Robin Moss, Eike Moritz Wülfers, Gunnar Seemann, Christian Wieners, and Axel Loewe. 2021. ‘Electro-Mechanical Whole-Heart Digital Twins: A Fully Coupled Multi-Physics Approach’. *Mathematics* 9 (11): 1247. https://doi.org/10.3390/math9111247.

Han, June-Chiew, Denis Loiselle, Andrew Taberner, and Kenneth Tran. 2021. ‘Re-Visiting the Frank-Starling Nexus’. *Progress in Biophysics and Molecular Biology*, Mechanobiology of the Cardiovascular System, 159 (January):10–21. https://doi.org/10.1016/j.pbiomolbio.2020.04.003.

Han, June-Chiew, Toan Pham, Andrew J. Taberner, Denis S. Loiselle, and Kenneth Tran. 2019. ‘Solving a Century-Old Conundrum Underlying Cardiac Force-Length Relations’. *American Journal of Physiology-Heart and Circulatory Physiology* 316 (4): H781–93. https://doi.org/10.1152/ajpheart.00763.2018.

Iribe, Gentaro, Toshiyuki Kaneko, Yohei Yamaguchi, and Keiji Naruse. 2014. ‘Load Dependency in Force–Length Relations in Isolated Single Cardiomyocytes’. *Progress in Biophysics and Molecular Biology*, Novel Technologies as Drivers of Progress in Cardiac Biophysics, 115 (2): 103–14. https://doi.org/10.1016/j.pbiomolbio.2014.06.005.

Kalda, Mari, Pearu Peterson, and Marko Vendelin. 2015. ‘Cross-Bridge Group Ensembles Describing Cooperativity in Thermodynamically Consistent Way’. *PLOS ONE* 10 (9): e0137438. https://doi.org/10.1371/journal.pone.0137438.

Kawel-Boehm, Nadine, Scott J. Hetzel, Bharath Ambale-Venkatesh, Gabriella Captur, Christopher J. Francois, Michael Jerosch-Herold, Michael Salerno, et al. 2020. ‘Reference Ranges (“Normal Values”) for Cardiovascular Magnetic Resonance (CMR) in Adults and Children: 2020 Update’. *Journal of Cardiovascular Magnetic Resonance* 22 (1): 87. https://doi.org/10.1186/s12968-020-00683-3.

Kuhtz-Buschbeck, Johann P., Angela Drake-Holland, Mark I. M. Noble, Brigitte Lohff, and Jochen Schaefer. 2018. ‘Rediscovery of Otto Frank’s Contribution to Science’. *Journal of Molecular and Cellular Cardiology* 119 (June):96–103. https://doi.org/10.1016/j.yjmcc.2018.04.017.

Landesberg, A. 1996. ‘End-Systolic Pressure-Volume Relationship and Intracellular Control of Contraction’. *American Journal of Physiology-Heart and Circulatory Physiology* 270 (1): H338–49. https://doi.org/10.1152/ajpheart.1996.270.1.H338.

Marx, Laura, Justyna A. Niestrawska, Matthias A.F. Gsell, Federica Caforio, Gernot Plank, and Christoph M. Augustin. 2022. ‘Robust and Efficient Fixed-Point Algorithm for the Inverse Elastostatic Problem to Identify Myocardial Passive Material Parameters and the Unloaded Reference Configuration’. *Journal of Computational Physics* 463 (August):111266. https://doi.org/10.1016/j.jcp.2022.111266.

Peirlinck, Mathias, Matthieu De Beule, Patrick Segers, and Nuno Rebelo. 2018. 'A Modular Inverse Elastostatics Approach to Resolve the Pressure-Induced Stress State for in Vivo Imaging Based Cardiovascular Modeling'. *Journal of the Mechanical Behavior of Biomedical Materials* 85 (September):124–33. https://doi.org/10.1016/j.jmbbm.2018.05.032.

Pironet, Antoine, Thomas Desaive, Sarah Kosta, Alexandra Lucas, Sabine Paeme, Arnaud Collet, Christopher G. Pretty, Philippe Kolh, and Pierre C. Dauby. 2013. 'A Multi-Scale Cardiovascular System Model Can Account for the Load-Dependence of the End-Systolic Pressure-Volume Relationship'. *BioMedical Engineering OnLine* 12 (1): 8. https://doi.org/10.1186/1475-925X-12-8.

Regazzoni, F., M. Salvador, P.C. Africa, M. Fedele, L. Dedè, and A. Quarteroni. 2022. 'A Cardiac Electromechanical Model Coupled with a Lumped-Parameter Model for Closed-Loop Blood Circulation'. *Journal of Computational Physics* 457 (May):111083. https://doi.org/10.1016/j.jcp.2022.111083.

Regazzoni, Francesco, Luca Dedè, and Alfio Quarteroni. 2018. 'Active Contraction of Cardiac Cells: A Reduced Model for Sarcomere Dynamics with Cooperative Interactions'. *Biomechanics and Modeling in Mechanobiology* 17 (6): 1663–86. https://doi.org/10.1007/s10237-018-1049-0.

———. 2020. 'Biophysically Detailed Mathematical Models of Multiscale Cardiac Active Mechanics'. Edited by Daniel A Beard. *PLOS Computational Biology* 16 (10): e1008294. https://doi.org/10.1371/journal.pcbi.1008294.

Rice, John Jeremy, and Pieter P. de Tombe. 2004. 'Approaches to Modeling Crossbridges and Calcium-Dependent Activation in Cardiac Muscle'. *Progress in Biophysics and Molecular Biology* 85 (2–3): 179–95. https://doi.org/10.1016/j.pbiomolbio.2004.01.011.

Salvador, Matteo, Francesco Regazzoni, Stefano Pagani, Luca Dede', Natalia Trayanova, and Alfio Quarteroni. 2022. 'The Role of Mechano-Electric Feedbacks and Hemodynamic Coupling in Scar-Related Ventricular Tachycardia'. *Computers in Biology and Medicine* 142 (March):105203. https://doi.org/10.1016/j.compbiomed.2021.105203.

Seemann, Felicia, Per Arvidsson, David Nordlund, Sascha Kopic, Marcus Carlsson, Håkan Arheden, and Einar Heiberg. 2019. 'Noninvasive Quantification of Pressure-Volume Loops From Brachial Pressure and Cardiovascular Magnetic Resonance'. *Circulation: Cardiovascular Imaging* 12 (1): e008493. https://doi.org/10.1161/CIRCIMAGING.118.008493.

Shimizu, Juichiro, Koji Todaka, and Daniel Burkhoff. 2002. 'Load Dependence of Ventricular Performance Explained by Model of Calcium-Myofilament Interactions'. *American Journal of Physiology-Heart and Circulatory Physiology* 282 (3): H1081–91. https://doi.org/10.1152/ajpheart.00498.2001.

Stella, Simone, Francesco Regazzoni, Christian Vergara, Luca Dedé, and Alfio Quarteroni. 2022. 'A Fast Cardiac Electromechanics Model Coupling the Eikonal and the Nonlinear Mechanics Equations'. *Mathematical Models and Methods in Applied Sciences* 32 (08): 1531–56. https://doi.org/10.1142/S021820252250035X.

Suga, H., K. Sagawa, and A. A. Shoukas. 1973. 'Load Independence of the Instantaneous Pressure-Volume Ratio of the Canine Left Ventricle and Effects of Epinephrine and Heart Rate on the Ratio'. *Circulation Research* 32 (3): 314–22. https://doi.org/10.1161/01.res.32.3.314.

Tomek, Jakub, Alfonso Bueno-Orovio, Elisa Passini, Xin Zhou, Ana Minchole, Oliver Britton, Chiara Bartolucci, et al. 2019. 'Development, Calibration, and Validation of a Novel Human Ventricular Myocyte Model in Health, Disease, and Drug Block'. Edited by José D Faraldo-Gómez, Naama Barkai, Thomas Hund, and Molly Maleckar. *eLife* 8 (December):e48890. https://doi.org/10.7554/eLife.48890.

Zappon, Elena, Matteo Salvador, Roberto Piersanti, Francesco Regazzoni, Luca Dede', and Alfio Quarteroni. 2024. 'An Integrated Heart–Torso Electromechanical Model for the Simulation of Electrophysiological Outputs Accounting for Myocardial Deformation'. *Computer Methods in Applied Mechanics and Engineering* 427 (July):117077. https://doi.org/10.1016/j.cma.2024.117077.

Dear Dr Regazzoni,

Re: JP-RP-2025-287282R1 "Elucidating the cellular determinants of the end-systolic pressure-volume relationship of the heart via computational modelling" by Francesco Regazzoni, Corrado Poggesi, and Cecilia Ferrantini

We are pleased to tell you that your paper has been accepted for publication in The Journal of Physiology.

The last Word (or similar) version of the manuscript provided will be used by the Production Editor to prepare your proof. When this is ready you will receive an email containing a link to Wiley's Online Proofing System. The proof should be thoroughly checked and corrected as promptly as possible.

Authors should note that it is too late at this point to offer corrections prior to proofreading. Major corrections at proof stage, such as changes to figures, will be referred to the Editors for approval before they can be incorporated. Only minor changes, such as to style and consistency, should be made at proof stage. Changes that need to be made after proof stage will usually require a formal correction notice.

All queries at proof stage should be sent to: TJP@wiley.com.

Following copyediting and typesetting the accepted version will be published online.

Yours sincerely,

Natalia Trayanova
Senior Editor
The Journal of Physiology

IMPORTANT POINTS TO NOTE FOLLOWING YOUR PAPER'S ACCEPTANCE:

If you would like to receive our 'Research Roundup', a monthly newsletter highlighting the cutting-edge research published in The Physiological Society's family of journals (The Journal of Physiology, Experimental Physiology, Physiological Reports, The Journal of Nutritional Physiology and The Journal of Precision Medicine: Health and Disease), please click this link, fill in your name and email address and select 'Research Roundup':
https://www.physoc.org/journals-and-media/membernews

• TRANSPARENT PEER REVIEW POLICY: To improve the transparency of its peer review process, The Journal of Physiology publishes online as supporting information the peer review history of all articles accepted for publication. Readers will have access to decision letters, including Editors' comments and referee reports, for each version of the manuscript as well as any author responses to peer review comments. Referees can decide whether or not they wish to be named on the peer review history document.

• You can help your research get the attention it deserves! Check out Wiley's free Promotion Guide for best-practice recommendations for promoting your work at: www.wileyauthors.com/eeo/guide. You can learn more about Wiley Editing Services which offers professional video, design, and writing services to create shareable video abstracts, infographics, conference posters, lay summaries, and research news stories for your research at: www.wileyauthors.com/eeo/promotion.

• IMPORTANT NOTICE ABOUT OPEN ACCESS: To assist authors whose funding agencies mandate public access to published research findings sooner than 12 months after publication, The Journal of Physiology allows authors to pay an Open Access (OA) fee to have their papers made freely available immediately on publication.

The Corresponding Author will receive an email from Wiley with details on how to register or log-in to Wiley Authors Services where you will be able to place an order.

• You can check if your funder or institution has a Wiley Open Access Account here: https://authorservices.wiley.com/author-resources/Journal-Authors/licensing-and-open-access/open-access/author-compliance-tool.html.

EDITOR COMMENTS

Reviewing Editor:

The Referees were satisfied with the new version of the paper.

REFEREE COMMENTS

Referee #1:

The reviewers appreciate the revisions made by the authors and agree that most of the comments have been adequately addressed.

However, we would like to point out a minor technical issue: while the DOI link for the Sagawa paper now correctly appears in the text, the embedded hyperlink in the PDF still directs to a non-existent DOI. This discrepancy may have been the cause of the initial issue encountered during the first submission.

Finally, regarding compliance with the journal's policy stating that "as in any experimental study, results from the model should add new scientific insights, and the model should be based on physiological data where parameters can be directly linked to experimental data," we refrain from assessing the authors' response and leave this to the discretion of the editor.

Referee #2:

All issues have been addressed.
